# A Context-Adaptive Hyperspectral Sensor and Perception Management Architecture for Airborne Anomaly Detection

**DOI:** 10.3390/s25196199

**Published:** 2025-10-06

**Authors:** Linda Eckel, Peter Stütz

**Affiliations:** Institute of Flight Systems, University of the Bundeswehr Munich, 85579 Neubiberg, Germany; peter.stuetz@unibw.de

**Keywords:** hyperspectral imaging, airborne anomaly detection, sensor management, perception management architecture, context-adaptive data processing

## Abstract

The deployment of airborne hyperspectral sensors has expanded rapidly, driven by their ability to capture spectral information beyond the visual range and to reveal objects that remain obscured in conventional imaging. In scenarios where prior target signatures are unavailable, anomaly detection provides an effective alternative by identifying deviations from the spectral background. However, real-world reconnaissance and monitoring missions frequently take place in complex and dynamic environments, requiring anomaly detectors to demonstrate robustness and adaptability. These requirements have rarely been met in current research, as evaluations are still predominantly based on small, context-restricted datasets, offering only limited insights into detector performance under varying conditions. To address this gap, we propose a context-adaptive hyperspectral sensor and perception management (hSPM) architecture that integrates sensor context extraction, band selection, and detector management into a single adaptive processing pipeline. The architecture is systematically evaluated on a new, large-scale airborne hyperspectral dataset comprising more than 1100 annotated samples from two diverse test environments, which we publicly release to support future research. Comparative experiments against state-of-the-art anomaly detectors demonstrate that conventional methods often lack robustness and efficiency, while hSPM consistently achieves superior detection accuracy and faster processing. Depending on evaluation conditions, hSPM improves anomaly detection performance by 28–204% while reducing computation time by 70–99%. These results highlight the advantages of adaptive sensor processing architectures and underscore the importance of large, openly available datasets for advancing robust airborne hyperspectral anomaly detection.

## 1. Introduction

Hyperspectral imaging has gained increasing attention in remote sensing over the past decade. Ongoing advances in sensor technology have led to smaller and more affordable systems that can now be readily deployed in unmanned aerial systems (UAS) [1,2]. Because they capture spectral information well beyond the visible range, hyperspectral sensors are particularly suitable for identifying visually concealed objects [3,4]. The additional spectral resolution also facilitates the discrimination of small targets and often improves detection performance [5,6,7,8]. Consequently, hyperspectral sensors are regarded as highly promising for localizing objects such as camouflage materials and unexploded ordnance (UXO), which has motivated extensive research into the deployment of hyperspectral imaging on small UAS platforms for reconnaissance purposes. Although other sensing modalities and multimodal fusion approaches also play an important role, this study concentrates on exploiting the benefits of hyperspectral data in this setting. Several approaches exist for performing such target localization. Spectral target detection is one common strategy, relying on the availability of known spectral signatures to detect this signature in the HSI [9,10]. Various procedures have been introduced so far, such as the well-known match filter, which uses statistical models, and more recent approaches based on deep learning, such as mask-driven dual autoencoders for target detection, or other advanced detection techniques [11,12,13]. Anomaly detection, in contrast, aims to identify outliers that markedly differ from the spectral background, thereby enabling detection without predefined specific target signatures [14,15]. In practice, individual data elements are compared to a neighborhood, which may consist of single pixels or small local regions [16,17]. For example, benchmark detectors such as the Reed–Xiaoli Detector use the spectral signature of the pixel and their neighborhood for comparison [18]. Other methods use deep neural networks that learn to reconstruct normality, but struggle to reconstruct the outlying anomalies. This allows them to detect anomalies through their remarkably higher reconstruction error compared to the neighborhood such as sparse priors constrained and deep convolutional autoencoders [19,20]. The stronger the deviation from the comparison, the more likely an element represents a target. This makes anomaly detection well suited for tactical reconnaissance tasks, particularly in cases where prior target knowledge is absent. At the same time, the method faces important challenges such as its sensitivity to changing image conditions [3,21,22]. Such variability is characteristic in real-world applications such as reconnaissance missions. Due to the dynamic nature of the environment, recorded image scenes are highly diverse and cannot be anticipated in advance. As a result, the performance of individual anomaly detectors may fluctuate significantly depending on the scene content, target properties, or algorithm characteristics. For reconnaissance missions, however, reliable detectors must achieve robust and consistent performance while remaining computationally efficient; both of which are critical under the resource constraints of onboard operation.

Within this context, two recurring tendencies can be identified across a wide spectrum of representative studies [19,20,23,24,25,26,27,28,29,30,31]. First, most works primarily aim to refine the detection algorithms themselves. Second, their reported improvements are typically validated on very small datasets, often only three to four single images, and rarely more than six. Given the substantial performance variability across different datasets, such evaluations provide only limited evidence of consistent reliability. Consequently, current studies provide little evidence that anomaly detectors can fulfill the robustness requirements of real-world airborne sensing or improve performance in general. Although no comprehensive survey explicitly summarizes these patterns, the cited works collectively represent widely referenced methods and illustrate general trends in the field. This raises the question of whether the recent improvements will genuinely translate into better performance in realistic conditions, or if they will simply replace one context-specific detector with another. In other words, it remains unclear whether state-of-the-art detectors will deliver the promised robustness and performance improvements once deployed in operational scenarios. This uncertainty motivates a shift in perspective: rather than continuing to optimize single detectors in isolation, one should ask whether a higher-level management of sensing and processing, already established in other modalities, can provide more reliable performance for hyperspectral data as well.

In this regard, one research direction proposes adapting processing strategies dynamically to the actual sensing conditions, aiming to stabilize detection performance despite changing environments. This idea has been investigated for other modalities, including airborne RGB, thermal, and LiDAR sensors, where both CNN-based object detection methods and classical approaches such as BLOB analysis have been used [32,33]. In these studies, the concepts of sensor management and perception management describe data-driven predictions of sensor performance and adaptive selection of processing procedures under varying environmental conditions. Such approaches extend beyond hardware-level control and establish a system-level perspective on sensor data processing. Reported results demonstrate that dynamic detector selection enhances performance, even with modern CNN-based recognition techniques. However, hyperspectral sensor technology has not yet been considered in this context. Examining whether such architectural principles can be applied to hyperspectral anomaly detection, and whether this enables more reliable performance than single detectors, therefore, appears highly valuable.

Motivated by these considerations, the present study seeks to improve the performance and robustness of hyperspectral anomaly detection by strategically deploying specialized detectors together with their parameter settings. To this end, we adapt the architectural idea of sensor and perception management to hyperspectral imaging and introduce the concept of hyperspectral sensor and perception management (hSPM). This constitutes a new sensor management architecture specifically designed for hyperspectral data processing, aiming for robust and consistent detection, which are key requirements for practical UAS-based anomaly detection. The hSPM enables the dynamic selection of specialized anomaly detectors according to the scene and context. Its effectiveness is evaluated using two extensive airborne datasets comprising hundreds of samples that exhibit seasonal and illumination differences as well as a variety of target types. These datasets represent a real-world reconnaissance application involving camouflage materials and unexploded ordnance (UXO). Moreover, its performance is benchmarked against state-of-the-art anomaly detectors, including the convolutional autoencoder-based Autonomous Hyperspectral Anomaly Detector (AUTO-AD) [20], the transformer- and autoencoder-based Gated Transformer for Hyperspectral Anomaly Detection (GT-HAD) [24], and the Collaborative Representation-based Detector CRD [34]. Results show that hSPM achieves consistently superior performance across the datasets and outperforms all baselines. Thus, the state-of-the-art anomaly detectors cannot provide robust domain generalization in our experiments, whereas the hSPM approach, with its automated dynamic selection of specialized detectors, demonstrates substantial potential for additional performance gains. The main contributions of this work are as follows:

- The development and public release of a new large-scale airborne hyperspectral dataset, designed to capture diverse environments, seasonal variation, and heterogeneous target types. This dataset provides a representative and openly available benchmark resource to support further research in airborne anomaly detection.
- The design of a unified hSPM architecture, integrating sensor context extraction, adaptive band selection, and perception management into a coherent system-level architecture tailored for hyperspectral data.
- The introduction of a context-based false-positive suppression mechanism, which leverages environmental knowledge to reduce spurious detections and increase robustness under variable airborne conditions.
- The comprehensive experimental evaluation across two airborne datasets, including camouflage materials and UXO targets, benchmarking the proposed hSPM against state-of-the-art anomaly detectors in terms of both detection accuracy and computational efficiency.

## 2. Methodology

In this section, we describe the proposed hyperspectral sensor and perception management methodology and the state-of-the-art anomaly detectors used for comparison. The hSPM architecture is structured into three core modules. First, the sensor context module extracts the spectral and environmental context of the scene, providing the basis for adaptive downstream processing. Second, the sensor management module selects the most informative spectral bands, thereby reducing dimensionality while retaining target-relevant information and improving efficiency. Third, the perception management module dynamically selects and configures the anomaly detector best suited to the current scene context. The evaluation of our approach against the state-of-the-art anomaly detectors is detailed in Section 4. The methodological foundation of hSPM builds, in part, on our earlier pre-studies [21,35], where specific components such as context extraction, band selection, and detector parameters were introduced and validated. The present work goes substantially beyond these contributions by

- introducing a fully developed perception management module that enables context-driven and dynamic detector selection under practical UAS constraints;
- integrating a novel context-based false-positive suppression mechanism to improve robustness;
- unifying all components into a coherent system-level architecture for the first time;
- conducting a comprehensive evaluation on a newly acquired, large-scale airborne dataset, with a focus on robustness in real-world reconnaissance scenarios.

### 2.1. Hyperspectral Sensor and Perception Management

**Figure 1:**
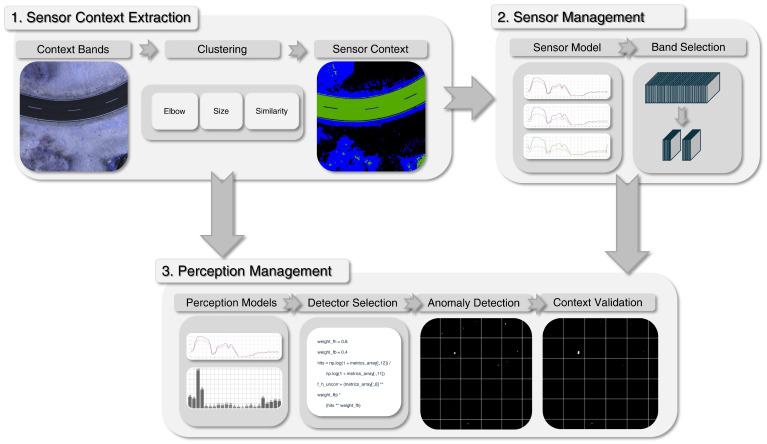
Overview of the Sensor and Perception Management architecture and its three main modules: (1) the Sensor Context, which extracts environmental areas (in this example: street—green, forest—blue, meadow—black); (2) the Sensor Management, which includes the Sensor Model and its predicted band performance for each area, followed by the Band Selection based on this performance; and (3) the Perception Management, which applies the Perception Model to predict detection performance (shown in purple) and subsequently selects the most suitable detector for Anomaly Detection and final Context Validation.

The proposed sensor and perception management (SPM) framework is composed of three core modules, illustrated in Figure 1. First, the sensor context module derives environmental and contextual information from the incoming hyperspectral image (HSI). This contextual knowledge enables subsequent modules, sensor management and perception management, to dynamically select the most informative spectral bands and the most suitable detection workflow. In this way, the architecture supports context-aware, resource-efficient, and robust anomaly detection under UAS constraints. The following subsections describe the specific procedures within each module in detail.

#### 2.1.1. Sensor Context

The sensor context module extracts representative spectral vectors that characterize the surrounding environment. Based on the current scene, dominant environmental regions are identified by clustering, providing the foundation for subsequent context-adaptive processing. As a first step, the incoming HSI is temporarily reduced to three fixed context bands, broadly distributed across the spectral range, see Table 1. These bands provide coarse spectral diversity and enable dimensionality reduction for efficient clustering. Their purpose is not to resolve fine-grained target details but rather to robustly distinguish broad environmental classes (e.g., forest, road, meadow) during the initial segmentation. Preliminary experiments with different selections of context bands showed negligible effects on clustering performance, given sufficient spectral spacing between the individual context bands.

**Figure 2:**
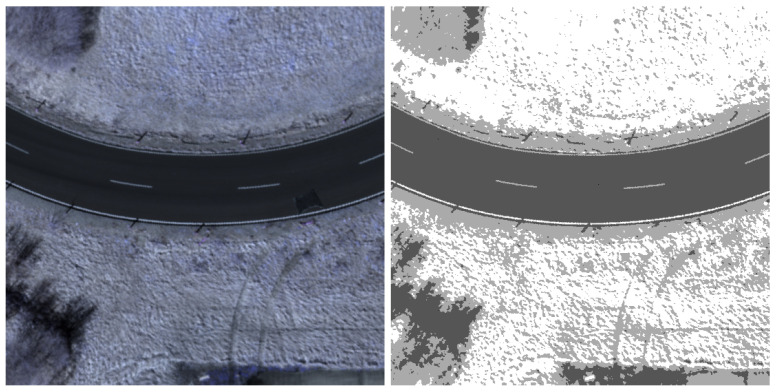
Example of sensor context extraction for a peri-urban winter scene from Dataset 1, showing context bands (**left**) and clustering output (**right**).

Subsequent clustering is performed using the well-known K-means algorithm, selected for its simplicity, fast convergence, and scalability on large-scale HSI data [36,37]. Since only a coarse segmentation of major environmental regions is required, more complex methods (e.g., DBSCAN, HDBSCAN, spectral clustering) would be unnecessarily costly. As demonstrated in prior studies [35], K-means yields semantically meaningful environmental clusters with high accuracy, confirming its suitability for this task. With the number of clusters $n_{k}$ predefined, the algorithm initializes by selecting random centroids $c_{k}$ ($k=1,\ldots ,n_{k}$) from shuffled data points. Each point is then assigned to the nearest centroid according to the squared Euclidean distance, and centroids are iteratively updated until convergence or a maximum number of iterations is reached [38]. To determine an appropriate $n_{k}$, the elbow method is employed [39,40,41], where the within-cluster sum of squares (WCSS) is analyzed to identify the point of diminishing returns. To accelerate this step, the spatial resolution of the context bands is temporarily reduced to a ground sampling distance (GSD) of 0.325 m. This gives the physical ground distance represented by a single pixel and means a spatial reduction of 20% for an input image with a GSD of 0.065 m, for example. To further ensure robust segmentation, two additional requirements are imposed: (i) as a spatial constraint, the clusters must exceed a minimum spatial size of 11 × 11 pixels at a GSD of 0.325 m. For reference, this absolute value is provided as reference for datasets with varying GSDs to enable the spatial constraints to be scaled accordingly, and (ii) as a spectral constraint, the spectral similarity between clusters is evaluated using the Normalized Cross Correlation (NormXCorr) [42]:

(1) $$\sum_{i=0}^{a}\left(\frac{\left(c_{i,l}-{\bar{c}}_{l}\right)\cdot \left(c_{i,m}-{\bar{c}}_{m}\right)}{\sigma_{c_{l}}\sigma_{c_{m}}}\right)$$

where *a* is the number of context bands, $\bar{c}$ denotes the mean of a centroid *c*, and $\sigma_{c}$ is the standard deviation. The variables *l* and *m* are the indices of the two clusters for which similarity is determined. If either criterion is not satisfied, $n_{k}$ is reduced until all conditions are met. Finally, clustering is performed on the full-resolution context bands using the optimized $n_{k}$. The resulting cluster label map is then used to mask the original HSI, after which average spectral vectors are extracted for each cluster over the complete spectral range of the dataset, thereby restoring the full dimensionality of the input image. These vectors, denoted $c_{env}$, represent the extracted sensor context and serve as input to both the sensor management and perception management modules. At this stage, the context bands are no longer used, and the original full spectral depth of the HSI is restored for subsequent processing. An example of this process is shown in Figure 2, illustrating the context bands (left) and corresponding clustering result (right).

#### 2.1.2. Sensor Management

The sensor management module estimates sensor performance based on the identified environmental context and the specific detection task for each spectral sensor band. Using the extracted spectral environmental vectors, a trained sensor model predicts how well the target group of interest can be distinguished from its surroundings. This prediction enables the identification and selection of spectral bands with the highest information value for anomaly detection under the given conditions. Together, the sensor model and the band selection mechanism constitute the sensor management module.

##### Sensor Model

The sensor model exploits the spectral environmental vectors $c_{env}$ to predict the deviation in potential targets from their background. Since anomaly detection assumes that objects of interest are spectrally unique compared to their environment, overall detection performance strongly depends on the degree of separability. This separability varies with environmental conditions and target properties, which in turn affects which spectral bands are most informative. As shown in [21,43], a targeted selection of the most meaningful bands allows a higher anomaly detection performance to be achieved while reducing computational demands. Consequently, dynamic and context-driven band selection is critical for robust performance. For this purpose, the sensor model is trained to predict the expected spectral deviation $v_{k,t}$, where *k* denotes the cluster index and *t* the target index, relative to the environment represented by the $n_{k}$ clusters:

(2) $$v_{k,t}=\frac{c_{{\mathrm{env}}_{k}}-\min\left(c_{{\mathrm{env}}_{k}}\right)}{\max\left(c_{{\mathrm{env}}_{k}}\right)-\min\left(c_{{\mathrm{env}}_{k}}\right)}-\frac{\bar{t_{s}}-\min\left(c_{{\mathrm{env}}_{k}}\right)}{\max\left(c_{{\mathrm{env}}_{k}}\right)-\min\left(c_{{\mathrm{env}}_{k}}\right)}$$

where $\bar{t_{s}}$ denotes the average target spectrum and $c_{{\mathrm{env}}_{k}}$ the environmental context vector of cluster *k*. Normalization with respect to $c_{{\mathrm{env}}_{k}}$ ensures stability across varying atmospheric conditions, allowing model training without explicit atmospheric correction for predicting $v_{k,t}$. The training set of targets is designed to cover a wide range of variations within the object group. This approach has already been explored in prior work, where it demonstrated stable predictions even for unseen targets and environments, underscoring its suitability for anomaly detection scenarios in which specific target knowledge is typically unavailable. In addition to the environmental context vectors, metadata such as target ID, target type, visual color, and season are also provided as inputs. The regression model is implemented as a Random Forest, chosen for its robustness, efficiency with small training sets, and ability to provide interpretable feature importance scores, properties that are particularly relevant for real-world applications. The training configuration of the sensor model for the varying test configurations in Section 4 are defined in Section 3. Overall, the sensor model enables an automated, context-driven prediction of sensor performance and facilitates robustness to varying image scenes without requiring manual intervention.

##### Sensor Band Selection

**Figure 3:**
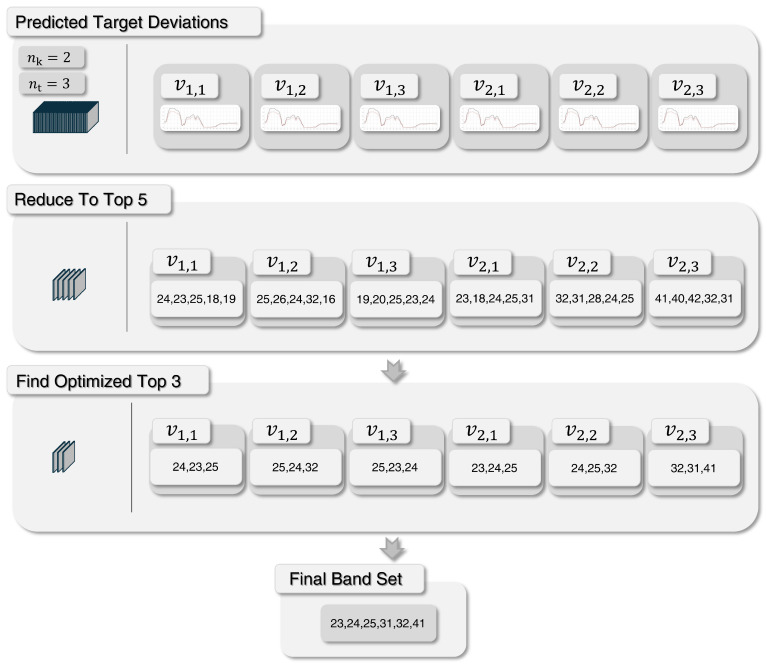
The Procedure for selecting the optimized band set in an example case. The example uses $n_{k}=2$ and $n_{t}=3$ and shows, for each target–environment combination $v_{k,t}$, the schematically plotted target deviation in red and the corresponding $c_{{\mathrm{env}}_{k}}$ in black. The band order of the determined deviation is descending.

The band selection procedure derives an optimal subset of spectral bands based on the predicted sensor performance by the sensor model. This ensures an efficient use of spectral information by reducing dimensionality while retaining the most discriminative features. The methodology operates as follows: For each environment–target combination (k,t), the sensor model predicts a target deviation vector $v_{k,t}$ across all spectral bands, see Figure 3. From these predictions, the five bands with the highest deviation are initially identified and sorted with ascending order. Afterwards, the top three bands for each environment–target combination are determined in favor of the increased reocurrence of the combinations. For this purpose, the top five pre-selected band candidates are examined for recurring bands and retained in ascending order of the target deviation. All $v_{k,t}$ that have 3 or more of theses consistently recurring bands will be assigned the 3 bands with the highest target deviation. If fewer than three recurring bands are found, additional bands are drawn from the 5 pre-selected candidates with the highest deviation scores. Thus, each environment–target pair (k,t) is represented by three selected bands and their corresponding predicted deviations from $v_{k,t}$, resulting in a maximum of $3\cdot n_{k}\cdot n_{t}$ unique bands across all combinations, where $n_{t}$ denotes the number of targets considered by the sensor model. Figure 3 illustrates this optimization procedure for selecting the optimal band set. This specific method of band selection allows the use of bands that provide high differentiability for targets and environments, while still achieving generally high differentiability across target and environment combinations, which prevents the selection of bands that negatively affect other target differentiabilities across the defined band set. The resulting band set is passed as input to the perception management module, where it provides a reduced yet highly informative spectral representation for subsequent anomaly detection. To account for the fundamentally different spectral–spatial properties of UXO and camouflage targets, the band selection procedure is executed separately for each target group. This yields two distinct band sets, one optimized for UXO, the other for camouflage, that are processed independently in the subsequent perception management stage.

#### 2.1.3. Perception Management

The perception management module extends the principle of sensor management to the level of anomaly detection. Its purpose is to predict and automatically select the most suitable anomaly detector for each input HSI based on the current scene context and target characteristics, see Figure 1. Detection performance strongly depends on both image conditions and target properties. This is because different anomaly detectors use different mathematical definitions of what constitutes an outlier, each based on a relative comparison to a chosen baseline. As a result, their responses vary with changes in background statistics or texture, which directly influence this comparison and thus the outcome of the detection. To address this challenge, our approach does not rely on a single detector. Instead, perception management exploits this variability by maintaining a pool of complementary detectors and using a trained perception model to predict detector performance and adaptively select the best configuration. Accordingly, perception management is divided into two sections. First, the pool of available anomaly detectors are introduced. Secondly, the perception model is proposed, which predicts the expected detection performance for all pool detectors and selects the optimal one. Due to the distinct spectral–spatial characteristics, separate detector pools and perception models are maintained for camouflage and UXO targets.

##### Detectors

The detector pool implemented in perception management is directly aligned with the characteristics of the evaluation dataset, which contains both large-area camouflage targets and small UXO objects under diverse environmental and seasonal conditions. To address these two fundamentally different target types, two detector pools are defined, see Table A1:

- For camouflage materials (large, often structures with varying color contrasts due to their camouflage texture), four algorithmic families are used—Local Reed–Xiaoli Detector (LRX), contour-based HDBSCAN (C-HDBSCAN), contour-based Normalized Cross Classification (C-NCC), and a bandpass filter, parameterized into 23 detector configurations.
- For UXO (small, point-like targets with limited spatial extent and often uniform contrast), a specialized LRX variant with adjusted parameters is applied, resulting in 7 additional detector configurations.

**Figure 4:**
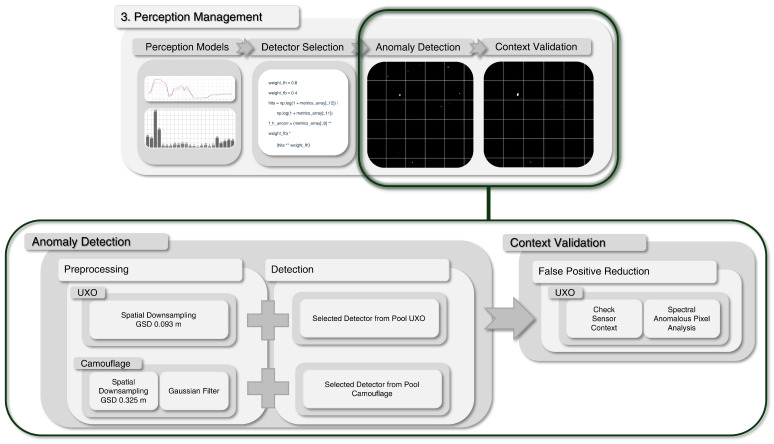
Workflow of the Perception Management with its 4 modules: (1) the Perception Models with the schematically plotted detection performance of the detector pool (shown in pink), (2) the Detector selection, (3) the Anomaly Detection and (4) the Context Validation. The Anomaly Detection module includes preprocessing of the selected sensor bands depending on the target group, followed by the actual anomaly detection process. The Context Validation module aims to reduce false positives from the upstream anomaly detection and is divided into the submodules Sensor Context Check and Spectral Anomalous Pixel Analysis.

These diverse detector pools ensure that both fundamentally different target types are addressed while providing complementary strengths across heterogeneous environments. The general workflow is illustrated in Figure 4, including preprocessing (downsampling and Gaussian filtering) and subsequent anomaly detection with a context valdiation for false positives reduction.

The detection processing of the camouflage material starts with a spatial downsampling and Gaussian filtering of the selected image band set as part of preprocessing. Spatial downsampling is performed to a GSD of 0.325 m, which represents a trade-off between preserving sufficient spatial resolution for detecting the targets and reducing computational load to ensure efficient processing while maintaining adequate coverage. Subsequently, a Gaussian filter smooths the image and reduces noise to enhance image quality affected by poor lighting conditions or bright reflections such as canopies. For this purpose, the filter is set with a Gaussian distribution of 0.5 and performed on the band set. The preprocessed images are then processed by one seleted anomaly detector out of the 23 camouflage detector configurations with the 4 algorithmic families. As a result of the strong methodological independence of the 4 algorithmic families, the performance of each detector varies greatly across changing image contexts in diverse image scenes. The methodology of the four algorithmic families is introduced below.

The Reed–Xiaoli Detector, first published in 1990 by [18], is one of the most popular anomaly detectors and a benchmark in HSI [44,45,46]. The detection algorithm models the background by a multivariate Gaussian distribution, assuming a homogeneous background. For this purpose, the detector compares a single pixel under test *x* with the background pixels *w* of a defined background $\mu_{w}$, which contains the image pixels within a specified inner window $w_{i}$ and outer window $w_{o}$ around *x*. If the defined window covers the entire image, the detector is referred to as the global Reed–Xiaoli Detector, otherwise, as the local one. Then, for each image pixel, the squared Mahalanobis distance $d_{M}^{2}$ is calculated to determine the level of abnormality to the background by

(3) $$d_{M}{\left(x\right)}^{2}={\left(x-\mu_{w}\right)}^{T}\sum_{w}^{-1}\left(x-\mu_{w}\right),$$

where $\mu_{w}$ is the mean vector of the background and $\sum_{w}$ the corresponding local covariance matrix that models the specified background. Once the LRX is performed, the detection map is converted into a binary detection mask by applying a percentile pctl that defines the required minimum percentage $d_{M}^{2}$ to be considered as an anomaly.

In addition to the LRX, the clustering-based detector C-HDBSCAN is implemented as part of the hSPM. This detector uses contour information, extracted by Felzenszwalb and Huttenlocher image segmentation, and the Hierarchical Density-Based Spatial Clustering of Applications with Noise (HDBSCAN). The HDBSCAN is a hierarchical density-based clustering algorithm that uses a minimum spanning tree (MST) to find the optimum clusters [47,48]. For that purpose, for all pairs of data points, the Bray–Curtis distance *d* is calculated, and the local density of the data by the mutual reachability distance $d_{r}$ is determined with

(4) $$d_{r}\left(F,G\right)=max\left\{d_{c}\left(F\right),d_{c}\left(G\right),d\left(F,G\right)\right\}$$

where *F* and *G* are the paired data points, and $d_{c}$ the core distance, which represents the minimum distance of the data points to its *l*-nearest neighbors and describes the data density around the data pairs. Using $d_{r}$, a mutual reachability distance graph is created, where all points are connected and weighted by their assigned distances. From this graph, an MST is computed, which directly connects all data points while minimizing the total sum of mutual reachability distances. This MST is the input for the hierarchical clustering that processes the MST with a varying level of detail in clustering. Subsequently, each cluster is analyzed with respect to its density, significance, and stability, and is selected based on the highest results. Hence, the process of hierarchical clustering can be controlled by setting a required minimum number of cluster pixels, called $c_{min}$. Some of the pixels may not be assigned to any of the clusters and are, therefore, identified as noise or anomalous data points. This exclusion is used to create a binary anomaly detection mask that is extended with contour information by Felzenszwalb and Huttenlocher image segmentation. The graph-based segmentation algorithm transforms the image pixels into nodes, connected through edges that represent their spatial distance and pixel value, defined as similarity [49]. Then, the algorithm varies the details of the segments, starting with a single segment for each pixel and terminating with assigning all pixels using a defined similarity threshold. The parameters $scale_{c}$ and $\sigma_{c}$ adjust the level of detail within this segmentation process. Finally, the resulting segments of the algorithm are checked for their size and transformed into a binary mask by thresholding the segments that exceed the maximum size, defined by $a_{max}$. A logical AND is then used to combine the segmentation mask with the result of the clustering step. HDBSCAN was implemented using [50,51] for the Felzenszwalb algorithm.

A contour-based Normalized Cross Classificator (C-NCC) based on [52] is also implemented alongside LRX and C-HDBSCAN. Here, the classifier that compares the image spectra to a given spectrum of interest is performed on the spectral environment vectors $c_{env}$ of the sensor context in Section 2.1.1. In this way, the NCC can be used for the detection of anomalous targets that deviate from the environment and remain unclassified. For this purpose, the NCC normalizes the given spectrum of interest and the spectral image in the first step. Subsequently, the NormXCorr is calculated for the spectrum of interest and each image pixel, providing a metric to measure their spectral similarities, see Equation (1). Hence, pixels that show generally low similarity below a defined percentile pctl across all spectral environment vectors are identified as anomalous or noise in a binary detection mask. Finally, this detection mask is combined with the contour information also extracted by the Felzenszwalb and Huttenlocher image segmentation algorithm using the same procedure as C-HDBSCAN.

The bandpass filter is the fourth detector implemented for the target group camouflage materials and isolates and identifies image pixels that differ significantly from the image background [53]. For this purpose, a passable signal range is defined by an upper and lower cutoff, called $l_{l}$ and $l_{h}$, which removes all signals outside the passable range. The bandpass filter creates, in the first step, a single 8-bit image from the spectral averaged input band set and determines the image center. This image center $r_{c}$ is defined by the pixel rows *H* and columns *W* of the image, which are combined by the theorem of Pythagoras. Hence, the defined cutoff values can be transformed into the value range of the image by multiplication with $r_{c}$ and the subsequent Fourier transform is prepared by creating a coordinate system for

(5) $$x_{i}=-\frac{H}{2}+i\cdot \frac{H}{H-1},\;i=0,1,\ldots ,H-1,$$

(6) $$y_{j}=-\frac{W}{2}+j\cdot \frac{W}{W-1},\;j=0,1,\ldots ,W-1,$$

(7) $$x_{ij}=x_{i},\;\forall j\in \left\{0,1,\ldots ,W-1\right\},$$

(8) $$y_{ij}=y_{j},\;\forall i\in \left\{0,1,\ldots ,H-1\right\}$$

where *x* and *y* are the arrays of the coordinate system used to calculate the Euclidean distances between them. With these distances and the determined image space cutoff values, the actual bandpass filter is created by a binary mask passing all distances within the cutoff range by

(9) $$f_{\mathrm{shifted}}\left(i,j\right)=f\left[\left(i+\frac{W}{2}\right)\;\mathrm{mod}\;\;W,\;\left(j+\frac{H}{2}\right)\;\mathrm{mod}\;\;H\right],$$

where *f_shifted_* is the shifted filter, which re-centers the low-frequency components to the center of the image before applying a 2-dimensional discrete Fourier transform. The product of the latter and the shifted bandpass filter is then transformed back into the initial image space by an inverse 2-dimensional discrete Fourier transform. In the last step, a percentage percentile pctl converts the image into a binary mask, depicting the extracted anomalous pixels. The parameter settings of all introduced detectors can be found in Table A1.

In addition to the detectors specialized for taller targets such as camouflage material, an LRX with an adjusted window size is used for the smaller targets UXO, see Table A1. Unlike the previously introduced detection procedure, the process of anomaly detection differs significantly and, besides a reduced downsampling rate, does not consider a Gaussian filter or any other detection algorithms other than the LRX, see Figure 4. This is due to the characteristics of the small target sizes, which require, on the one hand, a higher spatial resolution. On the other hand, the use of a Gaussian filter and the resulting image smoothing reduces the target differentiability for small targets and is not implemented due to this contradiction. Thus, only a spatial downsampling is implemented in addition to the detection algorithm LRX with variable parameter settings. While the LRX was the only detector that has shown higher detection rates with relatively low computational requirements, no other algorithmic family besides the LRX is implemented. In addition, the extensive postprocessing step context validation for reducing false positives is added subsequently to the UXO anomaly detection. The use of the statistically driven LRX for UXO detection causes a high false detection rate and must be reduced. To address this limitation, the next processing stage of the hSPM architecture, context validation, is introduced.

##### Context Validation

This module exploits the extracted sensor context and the predictions of the sensor model to systematically reduce false positives, as illustrated in Figure 1. In the first step of context validation, the detected anomalies are evaluated with respect to the extracted sensor context $c_{env}$ and the target deviations *v* predicted by the sensor model. The general idea is to leverage the cluster labels from the sensor context to filter out anomalous pixels associated with clusters that were subsequently classified as irrelevant. Hence, K-Means Clustering in the sensor context works with a fixed number of clusters; the algorithm assigns each data point to one of these clusters based on the smallest distance. Thus, each anomalous pixel is assigned to an environment cluster by the previous context extraction. At the same time, the sensor model has estimated for the targets of interest the expected target deviation $v_{k,t}$ to these clusters, and thus, for each target there exists a environment with the smallest deviation. Based on the K-means clustering method, all anomalies of interest must be assigned to the environment with the smallest distance in the context clustering. This means that environments without a minimum target deviation for any target should not contain anomalous pixels originating from actual targets but only false positives. Hence, all anomalies that were assigned to those environments in the context clustering are very likely not the target and are excluded from the detection map. In detail, all targets are shuffled to determine and list the environment with the assigned minimum target deviation, see Algorithm 1 Line 1 to 10. Subsequently, a binary mask is created by excluding all unlisted environments in the context clustering label map from Section 2.1.1, Line 11 to 12. This mask is then combined with the anomaly detection map using a logical AND operation, excluding all anomalies assumed to be false positives due to unassigned or irrelevant environmental clusters, Line 13.

**Figure 5:**
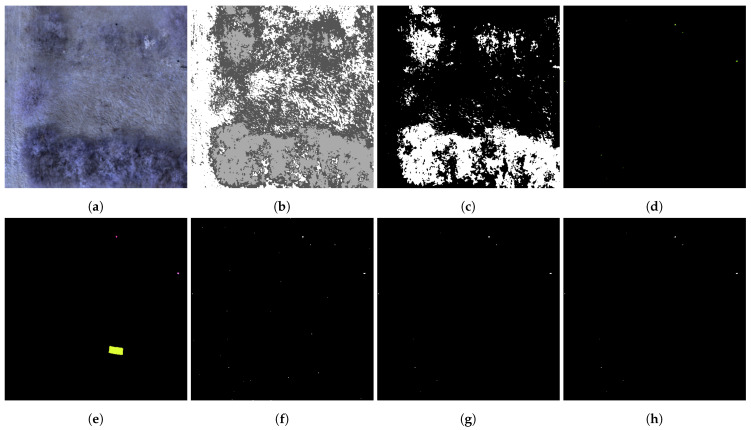
Example image from dataset 1 for reduced false alarm rate of UXO detection results by condsidering sensor context validation: (**a**) RGB image of raw HSI. (**b**) Extracted sensor context with three identfied areas (meadows in white, canopies in mid-grey and bushes and long grass in dark-grey). (**c**) Sensor context cluster with assigned minimum target deviation. (**d**) Clustered anomalous pixels. (**e**) Label mask with camouflage (yellow) and UXO (dark- and light-pink). (**f**) Raw UXO LRX anomaly detection map. (**g**) UXO LRX anomaly detection map with considered sensor context knowledge. (**h**) UXO LRX anomaly detection map after full context validation.

Figure 5c,g show an example of the improved detection performance with reduced false positives by considering the contextual knowledge of the sensor context. The image sample from dataset 1 shows two UXO and a camouflage net. Please note that the latter one is not detected by the LRX for UXO due to its selected window size for the much smaller UXO and is not considered as false negative. The detection of the camouflage material will be performed in parallel by the earlier introduced detectors specialized for camouflage.
**Algorithm 1** Check Sensor Context.**Require: detectionMap, envContextClusterLabel**, v, $n_{k}$, $n_{t}$**Ensure: detectionMapPostEnv, relevantEnvContextVecIndices** 1:  **relevantEnvContextVecIndices** =emptyList() 2:  **for** t=1 to $n_{t}$ **do** 3:        **tempDist**=emptyList() 4:        **for** k=1 to $n_{k}$ **do** 5:    Compute for target in environment: $\mu_{k,t}=\mathrm{mean}\left(v\left(k,t\right)\right)$ 6:    $\mathrm{tempDist}.append(\mu_{k,t})$ 7:        **end for** 8:        Determine *k* with min. **tempDist** for *t*: $k_{d_{min,t}}=\arg\min$(**tempDist**) 9:        $\mathrm{relevantEnvContextVecIndices}.append(k_{d_{min,t}})$10:  **end for**11:  $\mathrm{relevantEnvContextClusterLabel}=\left(\mathrm{envContextClusterLabel}\;.\in \mathrm{relevantEnvContextVecIndices}\right)$12:                           ▷ with .∈ elementwise containment check13:  detectionMapPostEnv=detectionMap.==relevantEnvContextClusterLabel14:                            ▷ with .== elementwise comparison15:  **return detectionMapPostEnv, relevantEnvContextVecIndices**

In addition to contextual postprocessing based on sensor context information, a second stage of false positive reduction is applied in the context validation: Spectral Anomalous Pixel Analysis. In this step, anomalous pixels are clustered according to their spectral signatures, grouping them into either potential anomaly targets or presumably spurious noise within the hyperspectral image (HSI). The underlying assumption is that, due to the initially high false alarm rate, the number of false positives remains larger than the number of true anomalous target pixels even after the first reduction stage. These false positives frequently originate from natural background variability, irregularities that can be interpreted as spectral noise. Because of their natural origin, many of these pixels are expected to share similar spectral characteristics and can thus be aggregated into a small number of large spectral clusters. These clusters can be distinguished from actual target clusters by comparing them to the sensor model-predicted target deviation *v*. This deviation is defined as the difference between the spectral signature of each anomalous pixel and the corresponding environmental context signature, as described in Equation (2). The assumption is that noisy clusters will exhibit greater dissimilarity to the predicted deviation than those clusters corresponding to actual targets. Therefore, noise clusters can be identified by their larger difference to the expected deviation and removed from the anomaly detection map, thereby lowering the false alarm rate. For each predicted target deviation $v_{k,t}$, anomalous pixels exhibiting the minimum distance to the sensor model prediction are identified, along with their associated context clusters. These clusters are then assumed to have a high likelihood of containing valid target information. In contrast, clusters not containing any pixel with minimum distance are assumed to be false positives and are excluded. This process is described in detail in Algorithm 2: First, the HDBSCAN algorithm is used due to its capability to form detailed spectral clusters, Line 4 to 6. The clustering process is governed by the parameters min_samples, set to 20, and true allow_single_cluster, which determine whether noisy or anomalous pixels can still form clusters, even in uniformly distributed data. If the number of anomalous pixels falls below min_samples, no cluster is created and the process is terminated. Afterwards, the target deviations for each anomalous pixel to the relevant context clusters are calculated, Line 7 to 12. Subsequently, distances between predicted target deviations and the calculated ones are determined using the minimum Euclidean distance and listed, Line 14. All HDBSCAN clusters that include pixels assigned a minimum distance are kept, Line 15. Finally, all non-relevant clusters are excluded from the anomaly detection map, Line 18 to 19. Since the accuracy of the procedure depends on the assumption that anomalies contain a large number of false positives, this assumption may no longer hold when only a few anomalies are present. Here, similarity and structuring by the HDBSCAN tend to be misleading. In such cases, the algorithm may produce overly granular clusters and inadvertently discard true positives. To address this issue, the proposed context validation is always applied in conjunction with the combined LRX configuration and its specific parameters. Each combination of detector configurations and context validation produces distinct detection results and must be taken into account by the downstream perception model. Figure 5d,h illustrate the clustered anomalous pixels and the resulting detection map. As shown in this example, the map following sensor context validation (g) already contains relatively few anomalous pixels due to the aforementioned limitations. Nevertheless, the clustering step is designed to address these challenges and results in the final refined detection map (h).
**Algorithm 2** Spectral Anomalous Pixel Analysis.**Require: HSI, envContextClusterLabel**, $c_{\mathrm{env}}$, v, $n_{k}$, $n_{t}$**Ensure: detectionMapFinal, relevantEnvContextVecIndices** 1:  **relevantHDBSCANClusterLabelIndex** =emptyList() 2:  Perform HDBSCAN clusterer on anomalous pixel: 3:        HDBSCANClusterLabel=HDBSCAN(HSI(detectionMapPostEnv)) 4:  **for** k=1 to $n_{k}$ **do**: 5:        HDBSCANClusterLabelEnv=HDBSCANClusterLabel(envContextClusterLabel.==k) 6:                             ▷ with .== elementwise comparison 7:        Calculate pixel deviations to corresponding $c_{\mathrm{env}}$ for each pixel signature $p_{s}$, 8:        where *u* denotes the pixel index and U the set of *u*: 9:  10:        **for all** u∈U **do**11:   ${\mathrm{pixelDeviation}}_{k}\left(u\right)=\frac{c_{{\mathrm{env}}_{k}}-\min\left(c_{{\mathrm{env}}_{k}}\right)}{\max\left(c_{{\mathrm{env}}_{k}}\right)-\min\left(c_{{\mathrm{env}}_{k}}\right)}-\frac{p_{s_{u}}-\min\left(c_{{\mathrm{env}}_{k}}\right)}{\max\left(c_{{\mathrm{env}}_{k}}\right)-\min\left(c_{{\mathrm{env}}_{k}}\right)}$12:        **end for**13:        **for** t=1 to $n_{t}$ **do**:14:   Get pixel index with min distance: $u_{k}^{*}\left(t\right)=\arg\min_{u\in U}{∥v_{k,t}-{\mathrm{pixelDeviation}}_{k}\left(u\right)∥}_{2}$15:   $\mathrm{relevantHDBSCANClusterLabelIndex}.append(\mathrm{HDBSCANClusterLabelEnv}\left(u_{k}^{*}\left(t\right)\right))$16:        **end for**17:  **end for**18:  $\mathrm{detectionMapFinal}=\left(\mathrm{HDBSCANClusterLabel}\;.\in \mathrm{relevantHDBSCANClusterLabelIndex}\right)$19:                           ▷ with .∈ elementwise containment check20:  **return detectionMapFinal**

##### Perception Model and Detector Selection

The perception model and the detector selection are the key components of the perception management and the instances that select the best-performing anomaly detector from the provided detector pools together with the corresponding pre- and postprocessing procedure. Each of the two target groups has its own perception model that is equal in meteorology and input features but trained only with data for the corresponding target group to adress the strong varying target characteristics. For this, the single perception models select the most suitable detector configuration for a given input band set from the band selection, which is also defined separately for each target group. The process begins by normalizing the input band set and converting it into an averaged single-band image in 8-bit format. From this image, a feature vector is extracted that serves as the input for predicting detector performance. Reliable prediction requires features that adequately capture the relationships between the targets, the image characteristics, and the properties of the detection algorithms.

The feature vector is constructed from Haralick and Local Binary Pattern (LBP) features, which describe texture, contrast, and entropy, and thus provide a representative abstraction of the scene. These features directly influence detector performance and allow the regression model to predict which detector is expected to perform best for a given scene. For the Haralick features, the average values across all four image directions are computed and concatenated to ensure directional invariance. The LBP features are likewise extracted in a directionally invariant manner, using a fixed radius of 111 and 5 sampling points, making them particularly effective for capturing coarser texture and contrast relevant for camouflage detection. In addition to these descriptors, the feature vector also includes the overall predicted mean target deviation as well as the minimum and maximum predicted target deviations from the sensor model. These values encode target-specific discriminability information for the model. The complete feature vector is then passed to a CatBoost regressor, which constitutes the perception model. Prediction proceeds in two steps: First, the regressor outputs a performance estimate for each detector in the pool with its pre- and postprocessing, expressed as a vector where each index corresponds to a specific detector. Secondly, based on these predicted values, the configuration with the highest score for detection performance is automatically selected. This detector performance is quantified using the newly proposed $f_{h}$-score, derived from the widely used $f_{\beta }$-score. The $f_{\beta }$-score is defined as follows:

(10) $$f_{\beta }=\frac{(1+\beta^{2})\cdot p\cdot r}{\beta^{,2}\cdot p+r}$$

where *p* is the precision, *r* is the recall, and β is the parameter that controls the weight of recall in relation to precision, set to 1.1 for a slight improvement in the overall detection sensitivity. However, the $f_{\beta }$-score is not a suitable metric for evaluating overall detection performance in scenarios with multiple anomalies, as it only considers the total number of correctly identified pixels, regardless of the individual targets present in the images. This can lead to a detection result with a single well-detected target being rated higher than a result where all targets are detected but less accurately. The latter is clearly preferable when applying HSI anomaly detection without needing contours for subsequent classification that can be performed using the spectral signature of individual pixels. The use of the AUC performance measurement, which determines the overall detector performance across all possible threshold values, also falls short here. The calculation includes a threshold range that may not be relevant in actual application scenarios, and it may also rank detectors with generally high performance above those that actually perform best within the relevant threshold range. To overcome these shortcomings, we propose a new metric, the $f_{h}$-score, which combines the $f_{\beta }$-score with the number of correctly detected targets in a scene into a single performance value. The $f_{h}$-score is defined as follows:

(11) $$f_{h}=f_{\beta }^{w_{f_{\beta }}}\cdot {\left(\frac{\ln(1+h_{d})}{\ln(1+h_{s})}\right)}^{w_{h}}$$

where $h_{d}$ is the number of correctly detected targets, $h_{s}$ is the total number of targets, and $w_{f_{\beta }}$ and $w_{h}$ are the weights (0.4 and 0.6, respectively). The logarithmic term prevents dominance by the number of targets and enables fair comparison across scenes. The weighted exponents ensure a unique mapping between input features and output scores, unlike a weighted sum. Finally, based on the predicted $f_{h}$-scores, the perception model automatically selects the detector configuration with the highest score for the given HSI scene, ensuring robust and adaptive detection performance. The training configuration of the two perception models used in this study are presented in Section 3.

### 2.2. Baseline Anomaly Detectors

In order to obtain a performance reference for hSPM, three additional anomaly detectors were processed using the HSI: the well-known statistically based CRD and the more advanced AUTO-AD and GT-HAD, representing the latest generation of anomaly detectors in HSI anomaly detection. The methodologies of these detectors are outlined below.

#### 2.2.1. Collaborative Representation-Based Detector

The CRD is one of the most popular statistical detectors in HSI and is, therefore, used to set a baseline detector against which hSPM’s performance can be compared. Similar to the statistical LRX, the CRD compares the local neighboring pixels *N* within an inner and outer window $w_{i}$ and $w_{o}$ to detect an outlier pixel *x* [34]. The assumption and main difference behind this detector is that all non-anomalous pixels can be represented by a weighted sum of the neighboring pixels, while outliers are completely different and cannot be represented as a linear combination. The concept is defined by

(12) $$\arg\min_{\alpha }{∥x-N\alpha ∥}_{2}^{2}+\lambda {∥\alpha ∥}_{2}^{2}$$

where α denotes the weight vector that allows the linear combination of *N* pixels to be transformed into a signal that is as similar as possible to the pixel under test *x*. The value λ sets the strength of this regularization term and can be used to balance between data fidelity (smaller values) and sparsity constraints. Solving the equations allows the residual res of the pixel under test *x* with respect to *N* to be calculated for the given α:

(13) $$res={∥x-N\alpha ∥}_{2}$$

The higher the res, the less cooperative the pixel is, and the higher the likelihood of the pixel being outlying.

#### 2.2.2. Autonomous Hyperspectral Anomaly Detector

In addition to the statistically based anomaly detector algorithms, the following Autoencoder-based AUTO-AD anomaly detector is implemented, see [54], and evaluated in the scope of this work. The AUTO-AD is an unsupervised, deep-learning-based anomaly detector that encodes pixel information into a lower-dimensional space and then decodes it back, reconstructing the original HSI data from its compressed version [20]. The assumption is that anomalous pixels are harder to reconstruct due to their uniqueness in the data, and thus have fewer representations in the data to learn. For this purpose, the AUTO-AD is iteratively trained on the input image, reconstructs it, and evaluates the reconstruction loss for image pixels after a defined number of iterations $i_{r}$ by

(14) $$L=\sum_{i=1}^{H}\sum_{j=1}^{W}{∥\left(x_{i,j}-{\tilde{x}}_{i,j}\right)*w_{a_{i,j}}∥}_{2}$$

where L is a loss function that measures the difference between the original x and reconstructed $\tilde{x}$ image, *H* and *W* are the image rows and columns, and $w_{a_{i,j}}$ is the weighting factor. Based on the idea that anomalous pixels have particularly large reconstruction errors in the early stages of iterations, the weight of these regions is reduced to force the prioritization of other regions in the reconstruction training, thus suppressing the reconstruction of likely anomalous pixels. The AUTO-AD terminates if the number of max iterations $i_{s}$ is reached or the total image reconstruction error σ is smaller than a user-defined threshold. While AUTO-AD does not require training data and adapts to the data characteristics during iterations, it also causes long processing times and high computational requirements. Nevertheless, the AUTO-AD algorithm is one of the latest and most popular algorithms for unsupervised anomaly detection in hyperspectral images and is, therefore, ideally suited for comparison with the proposed method.

#### 2.2.3. Gated Transformer for Hyperspectral Anomaly Detector

The GT-HAD also belongs to the group of autoencoder-based anomaly detection algorithms that use the reconstruction error to detect anomalies. However, the GT-HAD is newer than the AUTO-AD and achieves, based on [24], a higher detection performance than the AUTO-AD and is, therefore, used to compare against the hSPM detector. The main difference in comparison to AUTO-AD is that GT-HAD uses a Gated Transformer architecture instead of the convolutional autoencoder employed by AUTO-AD [20,24]. This is due to the fact that hyperspectral images are high-dimensional and transformer-based models are well suited to capture long-range spatial dependencies and content similarity between neighboring regions. Furthermore, one of the main advantages of GT-HAD is its ability to detect both point-like and area-like anomalies by breaking the image into smaller patches, which allows processing each region independently. This enables the detection of spatially extended anomalies, which often pose a challenge for global reconstruction approaches like AUTO-AD, as their size increases the likelihood of being incorporated into the background representation and thus being reconstructed. GT-HAD operates as follows: The input HSI is divided into overlapping patches, which are analyzed for their similarity to the surrounding regions. Based on this similarity, a Gated Transformer decides whether a patch is reconstructed by the background reconstruction branch or the anomaly-specific branch. The reconstruction is performed iteratively for each patch, and the training loss with respect to the input patch is evaluated as

(15) $$L\left(\theta \right)=\frac{1}{B}\sum_{j=1}^{B}{∥x_{i}-{\tilde{x}}_{i}∥}_{2}^{2}$$

where *B* are batch size per training iteration $i_{r}$ after the models parameters are updated with a defined learning rate η. The residual is then calculated by

(16) $$res\left(i\right)={∥x_{i}-{\tilde{x}}_{i}∥}_{2}^{2}\in R^{HxWxD}$$

where θ are the proposed learnable parameters of the net and *D* is the spectral depth. Subsequently, the residuals of all patches are stitched together in the final anomaly detection map. The implementation makes use of the original code repository from [55].

## 3. Evaluation

In the following section, the experimental setup for evaluating the detection performance of hSPM and its comparison with the state-of-the-art anomaly detection algorithms is presented. First, the HSI datasets as well as their configuration in train and test sets are introduced, followed by the detectors’ parameter grid testing to evaluate the best parameter and training settings on the defined train datasets.

### 3.1. HSI Datasets

**Figure 6:**
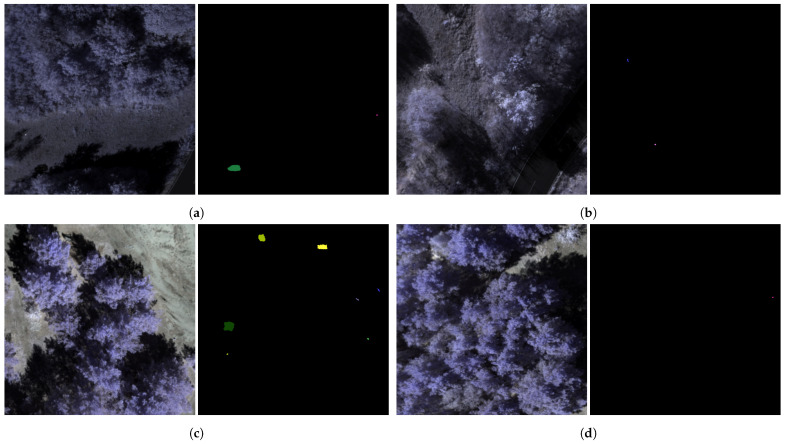
Randomly selected samples of Dataset 1 and 2 with their corresponding ground truths. (**a**) Sample of Dataset 1 with a mine (light-pink) and an improvised camouflage (green). (**b**) Sample of dataset 1 with a grenade (blue) and a mine (dark-pink). (**c**) Sample of dataset 2 with various UXO (purple, blue, light-green, mid-yellwow) and camouflage materials (dark green, light yellow, green-yellow). (**d**) Sample of dataset 2 with a mine (dark-pink).

**Figure 7:**
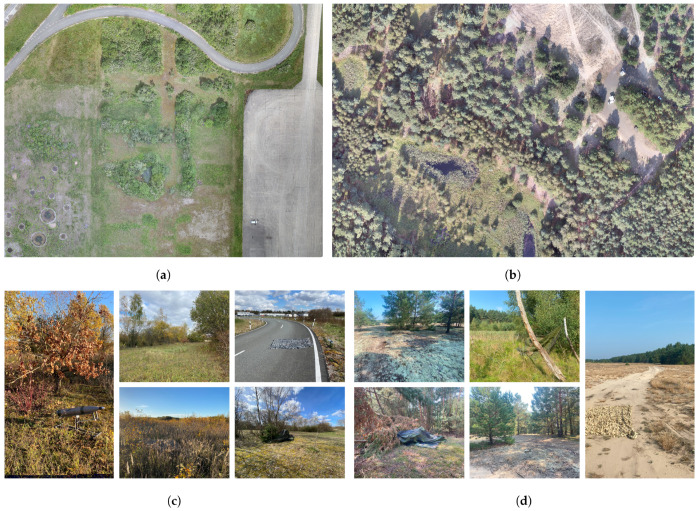
Airborne VIS-image of the two test sites and impressions of the experimental setup. (**a**) Test site 1 with meadow, deciduous forest, gravel, sand, and roads. (**b**) Test site 2 with coniferous forest and areas of swamp, moss, and sand. (**c**) Impressions of the experimental setup on test site 1. (**d**) Impressions of the experimental setup on test site 2.

**Figure A1:**
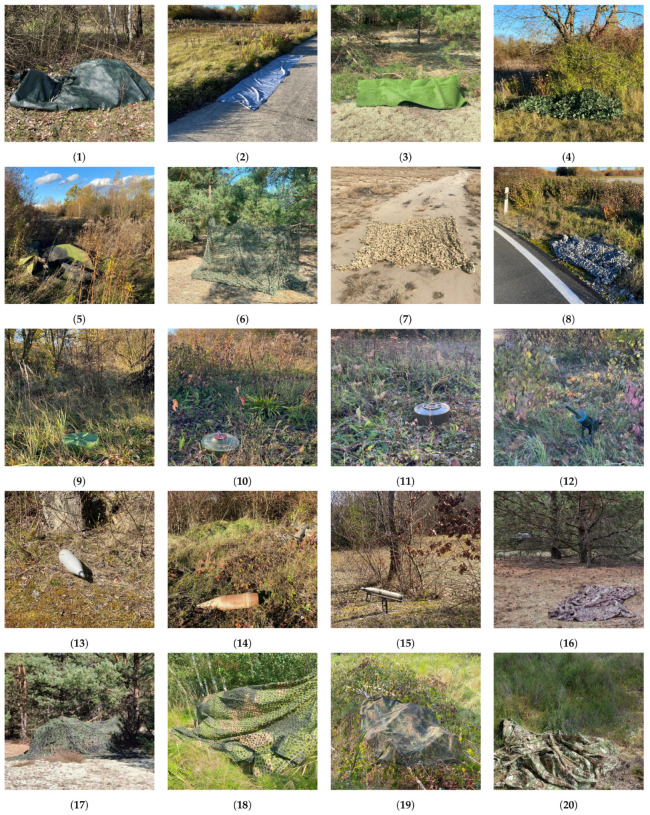

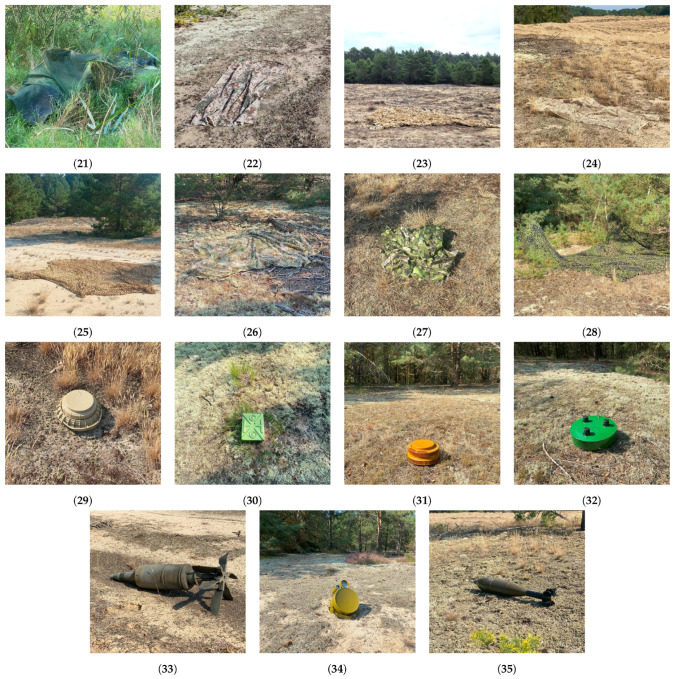
Visualization of the targets used in the HSI datasets.

The hyperspectral dataset used for testing the different anomaly detectors comprises a total of 1145 samples, covering a spectral range from 900 nm to 1700 nm with a spectral sampling interval of 3.5 nm and a spectral resolution of 8.0 nm. The data was acquired using a nadir-mounted Specim AFX17 sensor stabilized by a gimbal on a Freefly Alta-X UAS. It includes various scenes featuring camouflage materials and unexploded ordnance (UXO), recorded under different seasonal conditions at multiple test sites and from varying flight altitudes. The dataset is divided into two subsets reflecting these variations. Figure 6 presents randomly selected samples from both subsets, illustrating the broad diversity within the data. This variability, resulting from differences in season, location, targets, and target sizes, makes the dataset particularly suitable for comprehensive performance evaluations. Figure 7 provides an overview of the two test sites used. Dataset 1 comprises a total of 726 samples, including 331 samples with 421 depictions of UXO targets and 576 samples containing 659 depictions of camouflage materials, see Table 2. The targets used for image generation, labeled 1 to 15 and shown in Figure A1, offer a broad range of variations, resulting in a diverse target representation. It is worth noting that the UXO targets are dummies that replicate only the geometric characteristics. The samples were collected across all four seasons at an altitude of 60 m, yielding a GSD of 0.065 m. Data acquisition took place in a peri-urban environment featuring deciduous forest, grassland, roads, lakes, and concrete surfaces, referred to as test site 1.

Dataset 2 contains 419 samples and is significantly smaller than Dataset 1. It was designed to test the robustness of the anomaly detectors on a limited dataset that includes a wide variety of targets and conditions, offering only minimal training opportunities. Thus, the flight altitude for image capturing varies within the dataset between 50 m and 60 m. As defined in Table 3, Dataset 2 was captured in two different summer seasons: summer 1 at an altitude of 50 m with a GSD of 0.055 m, and summer 2 at an altitude of 60 m. The targets captured also vary. In summer season 1, targets 1 to 24 were used for image generation, whereas in summer season 2, targets 1 to 15, 17 to 19, and 24 to 34 were considered. In total, Dataset 2 contains 239 samples with 502 depictions of UXO and 380 samples with 735 depictions of camouflage materials. In addition to the target and altitude variation compared to Dataset 1, the test site also differs. Test site 2 consists of areas of swamp, moss, sand, and coniferous forest and simulates a typical anthropogenically undisturbed area. Overall, Dataset 2 complements Dataset 1 with more heterogeneous target and flight altitude conditions as well as a second test site for a higher departure sensor context across the datasets. This allows the detection performance and robustness of the hSPM to be determined in a broad scope.

For the evaluation of the proposed hSPM, two datasets were prepared. According to the presented methodology, both the sensor model and the perception model require training. Likewise, the anomaly detectors used for comparison also depend on HSI data to configure their parameters appropriately and achieve higher detection performance. For this purpose, the two datasets presented are split into training and test sets. The training sets are used to train the hSPM algorithm and to configure the parameters of the detectors for comparison, while the test sets are used to evaluate the actual detection performance with the corresponding training or parameter configuration of the train sets. For Dataset 1, which contains a uniform target configuration across varying seasons, the data was randomly split into 70% training data and 30% test data, while Dataset 2 represents a much more heterogeneous target configuration; Dataset 2 is used to create the test case of performance evaluation with unspecific training data, which is often the case in real application. Furthermore, it allows the evaluation of hSPM’s robustness and domain generalization, especially with respect to unspecific target information. As previously noted, a key advantage of anomaly detection over target detection is that it does not rely on specific target information. This property should also be demonstrated for hSPM, despite the fact that it is trained on HSI data containing target information. In addition, this setup enables a more meaningful comparison between hSPM and the other detectors, which require HSI data only for parameter configuration and, therefore, have no specific requirements regarding the data content. This test case, therefore, creates a scenario in which all detectors receive a training set with limited and unspecific data for optimization. For example, the GSD also varies across the data and is aligned to a uniform GSD of 0.065 m by nearest-neighbor interpolation. The resulting variation in the image dimensions *H* and *W*, therefore, must be considered by the detectors. To cover such a test scenario, all samples in Dataset 2 that contain targets 16–35 (see Figure A1) are assigned to the test split. This results in the following training and testing set configurations:

### 3.2. Training and Parameter Configuration

This section presents the evaluation of suitable detector parameters and training configurations based on the introduced HSI training datasets. First, the training setup for hSPM, including its sensor and perception models, is outlined and summarized in Table 4. For the sensor model, we adopted the robust parameter settings from the original publication, which have demonstrated stable performance across various scenarios. Additional tuning was deliberately avoided to reduce the risk of overfitting, particularly in Dataset 2, which contains only a limited number of training samples. Similarly, the perception models were trained using a configuration that prioritized generalizability. This allowed the same setting to be applied to both datasets, despite their substantial content and target characteristic variations, while avoiding overfitting. The hSPM training and testing is CPU-based and was conducted on a 12th Gen Intel i7-1260P CPU with 34 GB of RAM, Windows 11, and Python 3.10.14.

For the state-of-the-art detectors AUTO-AD, GT-HAD, and CRD, comprehensive grid testing was conducted on the training split data to evaluate the detector parameters that achieve the highest $f_{h}$-scores, as summarized in Table 5. As the computing platform, an NVIDIA DGX Station with seven NVIDIA H100 80 Gb HBM3 GPUs, an Intel Xeon Platinum 8480C CPU, and 1.72 Tb of RAM running Ubuntu 22.04 was used. The powerful NVIDIA DGX Station was selected due to the considerable computational demands of the state-of-the-art detectors. These detectors were implemented in accordance with their original publications to preserve their intended performance characteristics and to ensure a fair comparison with the proposed method. For the CPU-based CRD, the computational requirement is primarily driven by the spectral depth of the HSI data, consisting of 224 bands. Consequently, CRD is frequently used in combination with dimensionality-reduction techniques as preprocessing to mitigate computational load. Although such preprocessing steps can substantially influence detection performance, they are excluded in this study to enable a fair comparison with the other detectors that operate on the full spectral depth. Especially in the case of GT-HAD, processing the full spectral input led to considerable computational demands. Therefore, GT-HAD was evaluated solely under its primary configuration as proposed in the original publication. This decision was based on the considerable computational effort required by the default setting and the practical constraints of the evaluation framework. In contrast, both AUTO-AD and CRD underwent comprehensive grid testing across the training datasets, as defined in Table 5. This yielded optimized detector parameters achieving the highest $f_{h}$-scores for the training datasets, as shown in Table 6. These parameters were subsequently applied to the test datasets to evaluate the detectors’ performance.

Nevertheless, using an NVIDIA DGX Station as a computing platform for the three comparative detectors is out of range for an onboard application on a small reconnaissance UAS and is only used to generate an appropriate basis for comparison of the hSPM by using the original code and authentic implementation of the comparative detectors. To evaluate the detection performance in the context of tactical airborne reconnaissance tasks and their resource limitations, which is simulated by hSPM’s computing platform, we analyze the scalability of the CRD, GT-HAD, and AUTO-AD to the introduced use case. Assuming a comparable evaluation to the hSPM must be achieved, the scaled detector must be processed on the same platform in an application-oriented runtime of under 10 s, while retaining the detector’s authenticity and methodology without modifying the input HSI, such as PCA or MNF, or the algorithm itself. In the case of AUTO-AD, a successful scaling of the detector, defined as sAUTO-AD, was implemented by a slight adaptation of the network architecture and was also performed on the small computing platform. A parameter optimization was also performed on the training datasets using the parameter grid defined in Table 7, and the selected parameters in Table 6 are used to evaluate the detector’s performance on the test datasets for comparison.

## 4. Results

Section 4 is divided into two parts to analyze the perception performance of the hSPM and compare it with state-of-the-art detectors. In the first step, the newly introduced concepts of perception management and context validation are assessed with respect to their detection performance, potential, and contribution to overall improvement. Afterwards, hSPM is analyzed and compared with respect to the actual state-of-the-art hyperspectral anomaly detectors. Throughout this section, metrics annotated with an overbar represent averaged values across the corresponding dataset splits. Otherwise, the metrics represent the total score.

### 4.1. Performance Analysis Perception Management and Context Validation

For evaluation of the perception management and context validation performance, the sensor model is trained on the two train sets of Dataset 1 and 2. Using the trained sensor model, the theoretically achievable detection performance is determined under the assumption of a perfect selection of the best-performing detector on the test sets of Datasets 1 and 2. This theoretical performance is then compared to the actual detection performance achieved by the perception models trained on the corresponding training sets. The focus here is primarily on the $f_{h}$-score, based on which the detector performance was determined and optimized, and the hSPM was trained. The other performance values are presented for completeness and better understanding. Furthermore, the performance metrics are evaluated separately for the two target groups since the perception management architecture results in individual detection maps for each group. Table 8 shows the determined detection performances for the test sets of Dataset 1 and 2. The difference in the ${\bar{f}}_{h}$-scores between the theoretically reachable and the model-reached values is over 19% across both target groups and datasets, still indicating a great potential in the optimization of the model training process such as training configuration, data configuration, or feature selection. The largest performance gap is observed for the target group camouflage on Dataset 2 with a deficit of −38.87% to the theoretical maximum ${\bar{f}}_{h}$-score. For the UXO target group, the deficit for Dataset 2 is determined with −19.51%, closely matching the −20.18% of Dataset 1. Hence, the distances between the theoretical and the reached ${\bar{f}}_{h}$-scores are much smaller for the UXO across the datasets than for the camouflage target materials, with −23.84% and −38.87%. Thus, the possible potential for improvements in the camouflage perception model on Dataset 2 seems the highest.

However, the remaining losses in reached ${\bar{f}}_{h}$-scores are relatively close and datasets with their varying amount of training data and target variability. This is also supported by the normalized mean squared error (NMSE) of 0.0454 and 0.0563 for the UXO model trained on Dataset 1 and Dataset 2 with the proposed context validation, as well as 0.0414 and 0.0440 for the camouflage model, see Table 9. Hence, the results indicate a generally good adjustment of the models with respect to the datasets and targets, but an overall performance potential in the perception methodology should be further investigated to reduce the performance gap to the theoretically reachable ${\bar{f}}_{h}$-detection score. Despite the overall loss of prediction accuracy for Dataset 2, the goal of using general training parameters to achieve a robust and high detection rate across multiple datasets could be achieved. However, the overall detection performances on Dataset 2 are lower across all theoretical and actual performance scores and targets. This indicates that the targets in the test split of Dataset 2 are more difficult to detect using the perception management approach.

To enable a precise analysis of the efficiency and detection improvements achieved by the proposed context validation methodology, an additional experiment was conducted. For this purpose, the same sensor models from the previous evaluation, trained on the respective training sets of Datasets 1 and 2, were applied to their corresponding test splits. The theoretically achievable maximum ${\bar{f}}_{h}$-score was then calculated under three conditions for comparison: without context validation, with a sensor context check, and with the full two-stage context validation. In addition, the ${\bar{f}}_{h}$-scores of three trained UXO perception models were evaluated: one trained without context validation, one using the total ${\bar{f}}_{h}$-scores after the sensor context check, and one incorporating the scores obtained after completing the full two-stage context validation, including anomalous pixel analysis. Together with the corresponding NMSE values, this allows for an assessment of how well the trained models capture the varying detection performance across the different stages of the processing pipeline. As described in Section 2.1.3, each stage of the context validation introduces additional influencing factors, which may not be equally well captured by the selected features. Consequently, improvements in context processing do not necessarily result in a proportional increase in detection performance. All three perception models were trained using the same configuration specified in Table 4. The results of the experiment are presented in Table 10.

With focus on the theoretical ${\bar{f}}_{h}$-scores, the concept of context validation allows great performance improvements on Dataset 1. Especially the step of the sensor context check increases the detection performance significantly, mainly caused by an improved precision score $\bar{p}$. For Dataset 2, theoretical ${\bar{f}}_{h}$-score could also be improved by the context validation, but slightly lower. In particular, the sensor context check step leads to a decrease in the $f_{h}$-score due to lower detection sensitivity, $\bar{r}$ and $h_{t}$. However, the anomalous pixel analysis improves detection performance and can increase the total ${\bar{f}}_{h}$-score compared to raw detection results without context validation. This could be due to the characteristics of the two datasets. While Dataset 1 contains targets that are represented in the train as well as the test split data for evaluation, the train and test split data of Dataset 2 contain targets that are unknown for the sensor model that predicts the expected target deviation, which is also part of the context validation. Therefore, lower precision in the predicted target deviation by the sensor models also affects the effectiveness of the implemented context validation, which decreases from an NMSE of 0.0006 on the test split of Dataset 1 to 0.0035 on Dataset 2, see Table 9. This behavior can be fully replicated using the ${\bar{f}}_{h}$-scores achieved by the trained perception models. As in the previous case, a reduction in detection performance is observed in the first stage of context validation, but this loss is fully compensated for by the second processing stage, resulting in an overall increase in performance. Nevertheless, there was no training optimization performed in the three analyzed models, and the reached improved ${\bar{f}}_{h}$-scores can only be stated for the defined training configuration of the model. Within this configuration, the application of the sensor context validation methodology achieves higher detection performances and is, therefore, also applied to the subsequent studies. However, the application of the context validation must be carefully evaluated for utilization in test scenarios with low representation of the targets in the training data or a deviating training configuration. This can also be observed by the NMSEs of the Perception Models. The NMSE rises with the stages of context validation and from Dataset 1 to 2. Since the NMSE for the test split of Dataset 1 ranges from 0.0173 to 0.0310 to 0.0454 at the last stage of context validation, the Pixel Analysis, those for Dataset 2 are higher overall, at 0.0383, 0.0534, and 0.0563, see Table 9.

### 4.2. Benchmark Comparison

To gain an objective evaluation of the detection performance for the introduced methodology of hSPM, the anomaly detector is compared with the state-of-the-art detectors AUTO-AD, GT-HAD, and CRD as well as the more application-oriented sAUTO-AD. For this comparison, the detectors are performed on the test splits of Dataset 1 and 2 with their corresponding parameter settings and trained models based on the train splits. Since some of the detectors cannot distinguish between the two target groups, except for hSPM, a single detection mask is created. For this purpose, the detection masks of the detectors that differentiate between the target groups are combined with a logical AND operation into a single detection mask. Subsequently, the performance parameters are evaluated separately for each target group by excluding all labeled target pixels that are not of interest from the single detection masks. The results can be found in Table 11. The analysis also focuses on the ${\bar{f}}_{h}$-score, which was the crucial metric to evaluate the best-performing parameter settings and is the fundamental metric to define the absolute detection performance.

As a first step, the detection performance of the anomaly detectors for Dataset 1 is analyzed. Here, the detectors are trained and configured using a training dataset that well represents the data structure of the test dataset. Within this test scenario, hSPM gains the highest detection performance across the target groups: The achieved ${\bar{f}}_{h}$-scores of 0.2903 and 0.4280 for UXO and camouflage material, respectively, are significantly higher than the remaining scores gained by the other detectors. For example, the best ${\bar{f}}_{h}$-scores below are determined as 0.0955 and 0.2089, achieved by the AUTO-AD and CRD, respectively. This behavior can also be observed for the ${\bar{f}}_{1}$-scores, ${\bar{f}}_{\beta }$-scores as well as for precision and recall. Here too, hSPM achieves the highest score, with slight changes in the ranking of the detectors below it. In terms of the overall detected targets $h_{t}$, hSPM outperforms the other anomaly detectors when it comes to UXO. For the camouflage materials, the number of detected targets is much closer, led by an $h_{t}$ of 77.00. Here, the hSPM can only achieve the third-highest score of 74.50, but due to its significantly better recall and precision, it achieves a much higher overall detection performance while taking the shortest computational time per sample and using the fewest computational resources of any introduced platform. The sAUTO-AD and the GT-HAD have the lowest overall detection performance for both target groups and are ranked last.

In a second step, the detection performances of the anomaly detectors for Dataset 2 are evaluated. In this test case, the training dataset for parameter configuration and detector training represents an out-of-distribution scenario relative to the test set. This allows testing the domain generalization and robustness of the detectors with unknown targets. Furthermore, the clear separation and dissimilarity between the training and test sets enables a fair comparison between detectors that rely on supervised training and those that merely use the training data for parameter tuning. Within this test case, the hSPM also reached the highest overall detection performance across the two target groups with ${\bar{f}}_{h}$-scores of 0.1637 and 0.2545 for UXO and camouflage, respectively. However, the performance advantage has decreased, and the best below ${\bar{f}}_{h}$-scores are determined as 0.1236 for the UXO and 0.1987 for the camouflage by GT-HAD. Nevertheless, hSPM can obtain the lead also for the metrics ${\bar{f}}_{1}$, ${\bar{f}}_{\beta }$, $\bar{p}$, $\bar{r}$, and $\bar{t_{p}}$, while the scores for the parameter $h_{t}$ are ranked lower. The determined $h_{t}$ within the camouflage materials is the overall lowest. Here, the impact of the data distribution of Dataset 2 affects the selection of bands and detector settings as well as the performance of context validation, which is less accurate for unknown targets with new, unique characteristics. This is also reflected in the results previously introduced in Table 9 and Table 10. The lower accuracy of the sensor model’s predictions, as reflected by the increased NSME and the loss of performance in context validation compared to Dataset 1, highlights the importance of sensor model for hSPM’s overall performance. However, despite some metrics being lower, hSPM achieves the best overall performance as it scores highest across all target groups in the metric $f_{h}$-score, which is specifically defined to reflect the overall detection performance by the weighted impact of $h_{t}$, $f_{\beta }$, and thus also *p* and *r*. Furthermore, the supposed results in Section 4.1 show hSPM is still a great potential in detector optimization to gain the theoretically achievable detection performances for the datasets. These results indicate that, even under out-of-distribution conditions with previously unseen targets, hSPM shows clear advantages over the competing detectors and provides strong potential for robust anomaly detection.

However, the results also demonstrates that, within these tested configurations, the performance of state-of-the-art detectors is not necessarily robust or generalizable. This indicates that even high-performing, modern deep-learning-based detectors cannot necessarily replace the targeted use of specialized detectors, as implemented in the hSPM concept. Furthermore, the modular structure of hSPM enables the integration of such newer deep-learning-based detectors, allowing the approach to benefit from recent technological advances without excluding them. This has the potential to achieve wide performance gains. The fact that the contextual influence of environment, target, and detector characteristics should not be marginalized is also shown by the fluctuating optimal parameter settings in Table 6, which also support the fact that there is no sufficient basic generalization capability of the state-of-the-art detectors and that they cannot completely replace a targeted detector selection.

## 5. Discussion

However, it must be noted that the GT-HAD was only tested with a single parameter setting. Furthermore, some of the CRD’s selected parameter values lie at the boundaries of the tested parameter grid. Nevertheless, these experimental limitations are less of a methodological imprecision and more a reinforcement of the problem outlined in the Section 1: the strong contextual dependencies of the detectors, combined with limited studies on robustness and domain generalization. This underlines the relevance of a more adaptive approach such as hSPM. A closer analysis reveals the following: The GT-HAD exhibits significant differences in detection performance across the two datasets. For Dataset 1, GT-HAD is ranked among the lowest-performing detectors, whereas for Dataset 2, it ranks among the best. This divergence indicates a strong dependence of detection performance on the parameter configuration, thereby reinforcing the initial problem definition. In combination with the comparatively long runtimes per sample, this poses a practical challenge, especially since any performance-relevant optimization ultimately amounts to dataset-specific specialization. The need for such parameter tuning further highlights the limited domain generalizability and robustness of the method. As a result of the long computing duration per sample $h_{t}$, no comprehensive grid testing of GT-HAD’s parameters was conducted. A systematic evaluation within a practical timeframe would hardly have been feasible. Although alternative configurations might have led to better detection performance, possibly even surpassing that of hSPM, two aspects appear central:

1. A comprehensive grid search for optimal parameter settings is generally infeasible in real-world applications due to the high computational demand and is therefore rarely performed.
2. The results show that GT-HAD is not competitive without targeted and optimized parameter tuning, which effectively amounts to dataset-specific specialization. In contrast, hSPM achieves competitive detection performance using a generalized parameter setting, without such specialization. This limits the practical applicability and overall performance of GT-HAD in the context of the present study. Moreover, the very need for such specialization in GT-HAD underpins the motivation behind the hSPM approach: combining a set of anomaly detectors, each specialized for varying conditions, to achieve greater robustness, domain generalization, and performance, especially since few individual detectors exhibit genuine generalization. Hence, the observed behavior of GT-HAD reinforces the initially stated problem and the relevance of the hSPM approach.

However, an extended grid testing for parameter selections was conducted in the case of the CRD anomaly detector. Here, the maximum tested window size was set to 51 × 61 pixels at a GSD of 0.065 m, stated as appropriate to extract the background with respect to the actual sizes of the targets. While a slightly larger window size could theoretically offer marginal detection improvements, the runtime would increase significantly. Furthermore, given the already well-chosen configuration, the possibility of a notable performance improvement seems unlikely. Although, as already noted in the experiments, preprocessing techniques such as band selection or PCA could have been applied to reduce the computational load, such procedures have a significant impact on detection performance. Hence, an isolated evaluation of the detectors’ actual performance cannot be conducted. Since the other detectors use the full spectral depth of the data and are designed for this purpose, a comparison with a preprocessed CRD would be distorted. This also holds for hSPM. Unlike typical preprocessing approaches, which are characterized by a permanent data reduction, hSPM applies band selection only transiently as an integrated element, while retaining access to the full spectral depth in subsequent stages. Here too, extensive parameter testing would not be realistic for a more application-oriented use. The fact that hSPM achieves higher detection performance with a generically chosen training setting and a small number of samples, as defined in Table 2, compared to a carefully configured CRD, AUTO-AD, or sAUTO-AD, highlights the strength of the approach: Robust generalizability is achieved through the targeted combination of specialized detectors, eliminating the need for complex optimization. Although hSPM requires initial model training and thus labeled HSI, statistical detectors such as CRD and unsupervised detectors such as AUTO-AD also require labeled data in order to fit the detector parameters with respect to the actual detection task and environment, as demonstrated by the results. At the same time, the cost of training the hSPM appears to be lower and more efficient with respect to the shorter runtime per sample and the robust parameter settings. Even with generalized parameters, it achieved a level of detection performance in the conducted experiments that none of the comparison detectors could reach without further optimization. While approaches such as AUTO-AD and GT-HAD were introduced to gain more robust and domain-generalized hyperspectral detectors with unsupervised models, such as CNNs, to replace detectors with low domain generalization, the results suggest that the use of detectors with low domain generalization but high detection performance for defined conditions can also achieve great performance improvements: The targeted combination of context-specialized anomaly detectors, as realized in hSPM, can achieve significant performance improvements thanks to its heterogeneous structure and integrated architecture. This study has shown that it has the potential to surpass the robustness and domain generalizability of single anomaly detectors, such as AUTO-AD. At the same time, the modular architecture of hSPM can easily be extended to integrate the latest hyperspectral anomaly detectors. Therefore, the hSPM approach does not contradict the endeavors of GT-HAD, AUTO-AD, and others, but rather extends these endeavors.

Another aspect that needs to be noticed is the determined runtimes of the detectors. These measured times are not suitable for making absolute comparisons of the detectors with varying computational platforms and should be interpreted as a relative impression of the computational load. The varying computational platforms are a result of the great differences in the required computational resources. As the underlying use case, reconnaissance on small UAVs, involves limited onboard computing resources, the hSPM was designed to ensure a realistic runtime on a lower-performance, application-oriented platform. For this reason, the hSPM was evaluated on the originally intended test platform: a standard Dell office system, offering moderate CPU and GPU performance that reflects the resource constraints of typical onboard computing environments. However, it later became apparent that, given the high number of samples and the long processing times, the comparison detectors on this platform could only have been executed with considerably increased effort or restrictions on grid testing. The existing tests have already achieved implementation times of several weeks. To compare hSPM, the detectors for comparison were specifically selected because they are considered powerful representatives of current approaches in recent publications and their original implementations are publicly accessible and reproducible. Although detectors such as LREN, LRSR, and AED were considered initially, they were not evaluated further within the given framework due to their extremely high computing requirements and limited applicability. Substitution by other methods was also only possible to a limited extent, as adapting many of these methods to the target platform would have required significantly longer runtimes or extensive modifications. The AUTO-AD was the only exception, as it was the only one that could be transferred to the initial test platform. This scaled implementation was carried out under the requirement that the detector be executed on the same platform as hSPM, achieve an application-oriented runtime of less than 10 s, and remain consistent with the methodology and input HSI of the original publication. The detection performance achieved confirmed the authenticity of this approach. However, the other detectors (GT-HAD, CRD, and AUTO-AD) could not fulfill the scaling requirements. These were processed on the much more powerful NVIDIA DGX Station, and their determined runtimes are shown in the results. For this reason, it was considered best to run the remaining comparison detectors on a powerful NVIDIA DGX Station, in order to enable fair parameter evaluation. Where possible and methodologically justifiable, scaled detector implementations on the target platform were also carried out. This approach was considered closer to the application and more consistent methodologically than implementing all detectors on the DGX platform, particularly with regard to real-world feasibility in the UAV scenario. This differentiated approach did not result in any methodological disadvantages for the comparison detectors, and each detector could be evaluated under fair conditions and on its most suitable platform.

Many of the design decisions, such as the K-means algorithm for context clustering and the random forest regressor as the sensor model, were part of prior work. Therefore, this design could be questioned for the final hSPM architecture. The perception model bases its predictions on features that are the output of the sensor model and context extraction. The sensor model also works with input from the context extraction. Therefore, the high level of interference between context extraction, sensor models, and perception models has not yet been investigated and leads to uncertainty in the specific design decisions that may not be optimal. This can also be extended to the spectral dimensionality of the data. Due to the high spectral dimensionality of the HSI, the sensor model also predicts target deviations with high spectral depth. This complicates the prediction task because the training data remains unchanged in terms of samples and scenes. In contrast, reducing false positives using contextual knowledge greatly benefits from high spectral dimensionality and high differentiability. Therefore, further investigation is needed to understand these interferences and specify the break-even point of high spectral differentiability for reducing false positives and achieving higher accuracy in the sensor model’s predictions with lower prediction complexity.

Furthermore, the results in Section 4 highlight the importance of the accuracy of the sensor model’s predictions for the overall detection performance by hSPM. Therefore, further investigations are needed to explore this accuracy under varying conditions, and to develop techniques that stabilize the prediction accuracy or prevent the system from processing predictions with low reliability. One possible approach could be to implement quantile regression to evaluate the quantile intervals as a metric for uncertainty. This could allow adaptive processing procedures to be performed. These include bypassing context validation, performing more conservative context validation, or increasing the selected band set. The latter would improve the likelihood of adequate representation of unknown targets in the data.

## 6. Conclusions

In this paper, the hyperspectral anomaly detector hSPM was introduced and evaluated. A key feature of hSPM is its distinctive approach of combining several well-established detectors within a modular system architecture. Unlike other methods, this anomaly detector explicitly considers the characteristics of the image scene and the targets of interest, as well as the detector’s algorithm and its characteristics and how these affect its strengths and weaknesses. The fundamental assumption is that detectors behave differently under varying conditions and, therefore, require targeted deployment. This principle has received little attention so far, particularly in studies that rely on a limited number of standardized benchmark samples. By contrast, our evaluation explores detector performance under diverse and changing conditions. The results demonstrate that the proposed hSPM framework significantly outperforms current state-of-the-art detectors, even in scenarios where specific training data is unavailable and target characteristics must be assumed. In addition, hSPM operates with substantially shorter runtimes. These findings highlight the potential of hSPM architecture, showing that, with an optimal selection of detectors, the theoretical performance ceiling can be pushed even higher. Furthermore, our results suggest that improving individual detectors alone is often insufficient, as robustness and domain generalization can still be limited in practice. Additionally, the results suggest that improving individual detectors alone may not be sufficient, as resilience and cross-domain adaptation may still be limited in practice. Therefore, a promising approach is to combine detectors in a structured way within such a holistic architecture. The key benefit of the modular architecture is its versatility, which allows it to be used with a wide range of detection algorithms, including deep learning-based approaches. This versatility extends beyond the anomaly algorithms examined in this study.

## Figures and Tables

**Table 1:** Configuration of the three context bands.

Band	Wavelength
104	1294.53 nm
47	1095.17 nm
191	1602.35 nm

**Table 2:** Sample and target counts of the two datasets.

Parameter	Dataset 1		Dataset 2			
	Train	Test	Train		Test	
			50 m	60 m	50 m	60 m
UXO						
Samples	248	83	22	9	55	153
Target instances	309	112	23	21	68	390
Targets	9–15	9–15	9–15	9–15	all	all
Camouflage						
Samples	404	172	37	55	77	211
Target instances	459	200	46	68	176	445
Targets	1–8	1–8	1–8	1–8	all	all

**Table 3:** Configurations of the two datasets.

Parameter	Dataset 1	Dataset 2
test site	1	2
campains	4	2
altitude	60 m	50 and 60 m
seasons	summer to winter	summer
targets	1 to 15	1 to 35

**Table 4:** Selected training parameters of hSPM for Dataset 1 and 2.

Model	Parameter	Value
Sensor Model	Model	
	model type	random forest regressor
	toolbox	scikit-learn 1.6.0
	UXO & Camouflage	
	trees	100
	leaves	1
	split	2
	features	1.0
	fraction	0.0
	random state	42
Perception Model	Model	
	model type	catboost regressor
	toolbox	catboost 1.2.5
	UXO	
	iterations	700
	learning rate	0.1
	depth	8
	boarder count	170
	loss function	MultiRMSE
	Camouflage	
	iterations	800
	learning rate	0.1
	depth	8
	boarder count	170
	loss function	MultiRMSE

**Table 5:** Parameters for grid testing detectors CRD, AUTO-AD, and GT-HAD with the test set of each Dataset 1 and 2.

Detector	Parameters	Settings
CRD	*w_i_ / w_o_*	5/15, 9/21, 21/31, 31/41, 41/51, 51/61
	λ	$1\times 10^{-6}$
Auto-AD	σ	$1.5\times 10^{-7}$, $1.5\times 10^{-6}$, $1.5\times 10^{-5}$, 0.00015, 0.0015, 0.015
	*i_s_*	1000
	*i_r_*	100
	ϵ	0.1
	*C*	128
	*L*	5
GT-HAD	*i_s_*	150
	*i_r_*	25
	*B*	64
	η	0.001

**Table 6:** Selected parameters for the detectors.

Detector		Dataset 1		Dataset 2	
	Parameters	UXO	Camouflage	UXO	Camouflage
CRD	*w_i_/w_o_*	51/61	51/61	21/31	51/61
	λ	$1\times 10^{-6}$	$1\times 10^{-6}$	$1\times 10^{-6}$	$1\times 10^{-6}$
Auto-AD	σ	$1.5\times 10^{-6}$	0.0015	$1.5\times 10^{-7}$	0.0015
	*i_s_*	1000	1000	1000	1000
	*i_r_*	100	100	100	100
	ϵ	0.1	0.1	0.1	0.1
	*C*	128	128	128	128
	*L*	7	7	7	7
sAuto-AD	σ	$1.5\times 10^{-5}$	$1.5\times 10^{-5}$	$1.5\times 10^{-5}$	$1.5\times 10^{-5}$
	*i_s_*	60	60	60	60
	*i_r_*	2	2	5	2
	ϵ	0.03	0.07	0.03	0.05
	*C*	128	88	128	88
	*L*	7	7	5	7
GT-HAD	*i_s_*	150	150	150	150
	*i_r_*	25	25	25	25
	*B*	64	64	64	64
	η	0.001	0.001	0.001	0.001

**Table 7:** Parameters for grid testing with the scaled Auto-AD.

Parameters	Dataset 1	Dataset 2
σ	$1.5\times 10^{-5}$	$1.5\times 10^{-5}$
*i_s_*	50, 60	50, 60
*i_r_*	2, 5, 10	2, 5, 10
ϵ	0.03, 0.05, 0.07, 0.09	0.03, 0.05, 0.07
*C*	64, 88, 128	64, 88, 128
*L*	5, 7	5, 7

**Table 8:** Theoretical and reached performance of the perception management on the test sets of Dataset 1 and 2.

Target Group	Score	Theoretical	Reached	Relative [%]
Test Split Dataset 1				
UXO	$\bar{p}$	0.1162	0.0719	−38.12
	$\bar{r}$	0.3258	0.3117	−4.33
	${\bar{f}}_{1}$	0.1344	0.0920	−31.55
	$\bar{f}$ _ β _	0.1383	0.0959	−30.66
	*h_t_* [%]	83.04	72.32	−12.91
	${\bar{f}}_{h}$	**0.3632**	**0.2899**	**−20.18**
	$\bar{t}$*_p_* [s]	1.748	2.096	19.91
Camouflage	$\bar{p}$	0.3532	0.1998	−43.43
	$\bar{r}$	0.4592	0.3625	−21.06
	${\bar{f}}_{1}$	0.3542	0.2186	−38.28
	$\bar{f}$ _ β _	0.3575	0.2239	−37.37
	*h_t_* [%]	88.00	74.50	−15.34
	${\bar{f}}_{h}$	**0.5620**	**0.4280**	**−23.84**
	$\bar{t}$*_p_* [s]	1.852	1.782	−3.78
Test Split Dataset 2				
UXO	$\bar{p}$	0.0418	0.0192	−54.07
	$\bar{r}$	0.1895	0.2175	14.78
	${\bar{f}}_{1}$	0.0531	0.0315	−40.68
	$\bar{f}$ _ β _	0.0552	0.0338	−38.77
	*h_t_* [%]	50.87	48.69	−39.79
	${\bar{f}}_{h}$	**0.2009**	**0.1617**	**−19.51**
	$\bar{t}$*_p_* [s]	2.117	1.997	−5.67
Camouflage	$\bar{p}$	0.3081	0.1913	−37.91
	$\bar{r}$	0.2464	0.1557	−36.81
	${\bar{f}}_{1}$	0.2251	0.1362	−39.49
	$\bar{f}$_β_ (${\bar{f}}_{1}$)	0.2240	0.1354	−39.55
	*h_t_* [%]	64.57	38.49	−40.39
	${\bar{f}}_{h}$	**0.4163**	**0.2545**	**−38.87**
	$\bar{t}$*_p_* [s]	1.752	2.175	24.14

**Table 9:** NMSE values of the implemented sensor and perception models in hSPM for both datasets.

Type	Dataset 1	Dataset 2
Sensor Model		
UXO and Camo	0.0006	0.0035
Perception Models		
UXO—Raw	0.0173	0.0383
UXO—Sensor Context	0.0310	0.0534
UXO—Context Validation	0.0454	0.0563
Camo	0.0414	0.0440

**Table 10:** Theoretical and reached performance with consideration of additional contextual information for UXO on the test splits of Dataset 1 and 2.

	Score	Raw	Sensor Context	Pixel Analysis
Test Split Dataset 1				
theoretical	$\bar{p}$	0.0445	0.0963	0.1162
	$\bar{r}$	0.3206	0.3081	0.3258
	${\bar{f}}_{1}$	0.0582	0.1130	0.1344
	$\bar{f}$ _ β _	0.0606	0.1162	0.1383
	*h_t_* [%]	84.82	82.14	83.04
	${\bar{f}}_{h}$	**0.2332**	**0.3189**	**0.3632**
	$\bar{t}$*_p_* [s]	1.481	1.481	1.748
reached	$\bar{p}$	0.0276	0.0668	0.0719
	$\bar{r}$	0.2630	0.2524	0.3117
	${\bar{f}}_{1}$	0.0415	0.0854	0.0920
	$\bar{f}$ _ β _	0.0439	0.0887	0.0959
	*h_t_* [%]	70.54	66.96	72.32
	${\bar{f}}_{h}$	**0.1870**	**0.2615**	**0.2899**
	$\bar{t}$*_p_* [s]	1.481	1.481	2.096
Test Split Dataset 2				
theoretical	$\bar{p}$	0.0290	0.0350	0.0418
	$\bar{r}$	0.2696	0.1876	0.1895
	${\bar{f}}_{1}$	0.0424	0.1346	0.0531
	$\bar{f}$ _ β _	0.0447	0.0461	0.0552
	*h_t_* [%]	62.88	50.66	50.87
	${\bar{f}}_{h}$	**0.1972**	**0.1815**	**0.2009**
	$\bar{t}$*_p_* [s]	1.429	1.428	2.117
reached	$\bar{p}$	0.0195	0.0187	0.0192
	$\bar{r}$	0.1540	0.1449	0.2175
	${\bar{f}}_{1}$	0.0325	0.0732	0.0315
	$\bar{f}$ _ β _	0.0347	0.0305	0.0338
	*h_t_* [%]	40.30	37.99	48.69
	${\bar{f}}_{h}$	**0.1516**	**0.1319**	**0.1617**
	$\bar{t}$*_p_* [s]	1.427	1.429	1.997

**Table 11:** Compared detection performances for the hysperspectral anomaly detectors hSPM, AUTO-AD, sAUTO-AD, GT-HAD, and CRD on the test splits of Dataset 1 and 2.

Target Group	Score	hSPM	Auto-AD	sAuto-AD	GT-HAD	CRD
Test Split Dataset 1						
UXO	$\bar{p}$	0.0725	0.0141	0.0023	0.0109	0.0128
	$\bar{r}$	0.3117	0.1346	0.1492	0.0743	0.0700
	${\bar{f}}_{1}$	0.0924	0.0244	0.0046	0.0181	0.0202
	$\bar{f}$ _ β _	0.0962	0.0263	0.0050	0.0193	0.0212
	*h_t_* [%]	72.32	27.68	28.57	17.86	17.86
	${\bar{f}}_{h}$	**0.2903**	**0.0955**	**0.0502**	**0.0578**	**0.0541**
	$\bar{t}$*_p_* [s]	2.096	184.279	8.723	943.248	1281.303
Camouflage	$\bar{p}$	0.2012	0.0205	0.0217	0.0169	0.0227
	$\bar{r}$	0.3625	0.3095	0.3152	0.1223	0.3170
	${\bar{f}}_{1}$	0.2187	0.0376	0.0398	0.0284	0.0413
	$\bar{f}$ _ β _	0.2239	0.0408	0.0432	0.0304	0.0448
	*h_t_* [%]	74.50	77.00	74.00	52.50	78.50
	${\bar{f}}_{h}$	**0.4280**	**0.2060**	**0.2027**	**0.1346**	**0.2089**
	$\bar{t}$*_p_* [s]	1.782	20.540	7.216	943.248	1281.303
Test Split Dataset 2						
UXO	$\bar{p}$	0.0201	0.0064	0.0012	0.0091	0.0006
	$\bar{r}$	0.2285	0.1953	0.2882	0.2176	0.1650
	${\bar{f}}_{1}$	0.0339	0.0122	0.0023	0.0172	0.0012
	$\bar{f}$ _ β _	0.0365	0.0134	0.0026	0.0187	0.0014
	*h_t_* [%]	48.69	48.69	60.70	43.45	40.83
	${\bar{f}}_{h}$	**0.1637**	**0.1106**	**0.0638**	**0.1236**	**0.0368**
	$\bar{t}$*_p_* [s]	1.997	171.113	8.081	829.026	132.409
Camouflage	$\bar{p}$	0.1915	0.0159	0.0161	0.0191	0.0159
	$\bar{r}$	0.1557	0.1300	0.1316	0.1521	0.1526
	${\bar{f}}_{1}$	0.1362	0.0265	0.0269	0.0320	0.0271
	$\bar{f}$ _ β _	0.1355	0.0283	0.0288	0.0343	0.0291
	*h_t_* [%]	38.49	71.01	74.24	75.68	75.68
	${\bar{f}}_{h}$	**0.2545**	**0.1825**	**0.1851**	**0.1987**	**0.1834**
	$\bar{t}$*_p_* [s]	2.175	18.740	7.333	829.026	1204.915

## Data Availability

The datasets presented in this article can be found under the DOI: https://doi.org/10.5281/zenodo.16737351.

## Abbreviations

The following abbreviations are used in this manuscript:

AUTO-AD	Autonomous Hyperspectral Anomaly Detector
C-HDBSCAN	Contour-Based Hierarchical Density-Based Spatial Clustering of Applications with Noise
C-NCC	Contour-Based Normalized Cross Classification
CNN	Convolutional Neural Network
CRD	Collaborative Representation-based Detector
GSD	Ground Sampling Distance
GT-HAD	Gated Transformer for Hyperspectral Anomaly Detector
HSI	Hyperspectral Image
LRX	Local Reed–Xiaoli Detector
MST	Minimum Spanning Tree
NMSE	Normalized Mean Squared Error
NormXCorr	Normalized Cross Correlation
hSPM	Hyperspectral Sensor and Perception Management
UASs	Unmanned Aerial Systems
UXO	Unexploded Ordnance
WCSS	Within-Cluster Sum of Squares

## List of Symbols

The following symbols are used in this manuscript:

Variable	Description
*a*	Number of context Bands
$a_{\max}$	Maximum segment size
α	Weight vector for linear combination in CRD
*B*	Batch size per training
*C*	Number of neurons per hidden layer (GT-HAD)
*c*	Spectral cluster centroid
$c_{\min}$	Minimum cluster size
$c_{\mathrm{env}}$	Spectral environment vectors
*D*	Spectral Depth
*d*	Bray–Curtis dtistance
$d_{c}$	Core distance based on *d*
$d_{r}$	Mutal reachability distance based on *d*
$d_{M}$	Mahalanobis distance between *x* and $\mu_{w}$
ϵ	Regularization noise standard deviation
η	Learning rate for network optimization
*F*	Data point for calculating $d_{r}$
$f_{1}$	$f_{1}$-score
$f_{\beta }$	$f_{\beta }$-score
$f_{h}$	$f_{h}$-score
$f_{shifted}$	Shifted filter for applaying 2-dimensional discrete Fourier transform
*G*	Data point for calculating $d_{r}$
*H*	Number of image rows (height)
$h_{d}$	Number of correctly detected targets
$h_{s}$	Total number of targets in data/sample
$h_{t}$	Divisor of $h_{d}$ to $h_{s}$
$i_{r}$	Number of iterations before loss evaluation
$i_{s}$	Maximum number of training iterations
*k*	Cluster index (environment index)
L	Reconstruction loss
$l_{h}$	Higher cutoff value for bandpass filter
$l_{l}$	Lower cutoff value for bandpass filter
λ	Regularization parameter in CRD
$\mu_{w}$	Mean background vector of window *w*
*N*	Matrix of neighboring pixel vectors
$n_{k}$	Number of clusters of type *k*
$n_{t}$	Number of targets in the scene
*p*	Precision score
pctl	Percentile threshold decision masks
*r*	Recall score
res	Residual
$r_{c}$	Image center
$scale_{c}$	Segmentation scale parameter
σ	Reconstruction error threshold
$\sigma_{c}$	Segmentation standard deviation
$\Sigma_{w}$	Covariance matrix of window *w*
*t*	Target index
$t_{p}$	Processing time per image
$t_{s}$	Target spectrum
*v*	Target deviation
*W*	Number of image columns (width)
*w*	Background Window
$w_{a_{i,j}}$	Weighting factor for pixel reconstruction loss in AUTO-AD
$w_{f_{\beta }}$	Weighting factor for weightig $f_{\beta }$ in $f_{h}$
$w_{h}$	Weighting factor for weightig target hits in $f_{h}$
$w_{i}$	Inner background window
$w_{o}$	Outer background window
*x*	Pixel under test
x	Original image pixels
$\tilde{x}$	Reconstructed image pixels
Note: An overbar (e.g., $\bar{p}$) denotes the mean value of the respective quantity.	

## Appendix A. Parameters and Settings of the hSPM Detectors

**Table A1:** Parameters and settings of the hSPM detectors.

Detector	Parameter	Setting							
UXO									
LRX	ID	1	2	3	4	5	6	7	
	*pctl*	0.9690	0.9790	0.9890	0.9990	0.9995	0.9997	0.9999	
	*w_i_*	15	15	15	15	15	15	15	
	*w_o_*	31	31	31	31	31	31	31	
Camouflage Materials									
LRX	ID	1	2	3	4				
	*pctl*	0.9840	0.9890	0.9940	0.9990				
	*w_i_*	51	51	51	51				
	*w_o_*	101	101	101	101				
C-HDBSCAN	ID	5	6	7	8	9	10		
	*c_min_*	15	15	15	15	15	15		
	*a_max_*	70	70	70	70	70	70		
	*scale_c_*	375	375	375	500	500	500		
	*σ_c_*	0.3	0.4	0.5	0.4	0.5	0.6		
C-NCC	ID	11	12	13	14	15	16	17	18
	*pctl*	0.40	0.60	0.40	0.60	0.40	0.60	0.40	0.60
	*a_max_*	75	75	75	75	75	75	75	75
	*scale_c_*	375	375	75	375	500	500	500	500
	*σ_c_*	0.3	0.3	0.4	0.4	0.5	0.5	0.6	0.6
Bandpass	ID	19	20	21	22	23			
	*pctl*	0.995	0.995	0.995	0.995	0.995			
	*l_l_*	0.02	0.02	0.02	0.02	0.04			
	*l_h_*	0.06	0.07	0.08	0.09	0.09			
